# In‐Process Magnetization for 3D Printing of Magnetorheological Elastomer with Heterogeneous Magnetic Profile for Anisotropic Actuation

**DOI:** 10.1002/advs.76045

**Published:** 2026-06-12

**Authors:** Phillip Glass, David Hassouna, Udena Epitawala Arachchige, Hyeon Yun Jeong, Sung Hyun Park, Daeha Joung

**Affiliations:** ^1^ Department of Physics Virginia Commonwealth University Richmond Virginia USA; ^2^ Clean Energy Transition Group Korea Institute of Industrial Technology (KITECH) Jeju‐si, Jeju Special Self‐Governing Province Republic of Korea

**Keywords:** 3D printing, magneto‐active materials, programmable magnetization, spatially programmable actuation

## Abstract

Soft magnetic actuators have gained significant interest for applications in minimally invasive medical robots, artificial muscles, soft robotic manipulators, and wearable bioelectronic interfaces, yet their functionality remains fundamentally limited by current magnetization strategies. To this end, a novel in‐process printing and magnetization strategy with spatial and dynamic control of an external magnetic field during printing is developed to fabricate magnetorheological elastomers with fully customizable three‐dimensional (3D) magnetization profiles. This method allows localized magnetic domain alignment in arbitrarily programmed orientations within a solid, enabling anisotropic actuation at micron to millimeter scales. The proposed method is highly sensitive to curing kinetics, material viscosity, and magnet positioning, which are characterized theoretically, experimentally, and in simulation. Structures magnetized in this way offer robust strain‐sensing, information‐encoding, and bio‐inspired heterogeneous actuation capabilities. Demonstrations highlight this versatility, including a dragonfly with oppositely magnetized wings for tunable resonant actuation, an octopus‐inspired swimmer whose magnetized legs reproduce aquatic locomotion, and a serpentine catheter with high degrees of freedom across 6 magnetic nodes. Together, these advances establish a versatile platform for designing magnetically responsive systems that couple programmable anisotropic actuation with biological complexity.

## Graphical abstract

Fully programmable, in‐process 3D magnetization integrated with multi‐material printing enables soft magnetic systems with precise actuation and sensing. Discrete and continuous magnetization profiles drive bending, morphing, and locomotion, demonstrated through strain‐sensing elements, dragonfly‐inspired wings, octopus‐like tentacles, and a serpentine catheter with high degrees of freedom, establishing a versatile platform for soft robotics, adaptive devices, and bioinspired materials.

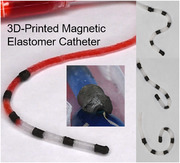

## 1 Introduction

Soft sensing and actuating systems provide a foundation for programmed interaction with the environment, enabling adaptive movement and perception [1, 2, 3, 4]. These capabilities are made possible by stimuli‐responsive materials, which either transduce external inputs into electrical signals (sensors) or physically respond to stimuli such as thermal, pneumatic, chemical, or electrical cues (actuators) [5, 6, 7, 8, 9]. Within this broad class, ferromagnetically responsive composites are particularly compelling due to their instantaneous, untethered response at a distance and their ability to be programmed for complex three‐dimensional (3D) sensing and actuation [10, 11, 12, 13]. Such programmable elastomeric ferromagnetic solids are relevant to diverse applications, including targeted drug delivery [14, 15], minimally invasive surgical tools [16, 17, 18, 19], implantable devices [20], soft robotics [19, 21, 22, 23, 24], flexible electronics [19, 25], and advanced sensing technologies [26, 27, 28, 29]. Achieving these functions, however, requires precise control of the internal magnetic moment distributions within the material during magnetization. Thus, the magnetization strategy fundamentally defines the complexity and performance of the resulting actuation.

Early approaches relied on uniform, post‐process magnetization of soft composites, applied after the manufacturing step [14, 30, 31]. In this method, already fabricated structures were exposed to a strong impulse field (1–3 Tesla), producing a unidirectional one‐dimensional (1D) magnetization profile. A variation involved mechanically deforming the composite into a desired shape, fixing it, and then magnetizing it. After release, the material recovered its original geometry while retaining a non‐uniform two‐dimensional (2D) profile, enabling heterogeneous bending under an external field [22, 32, 33]. While effective for simple, thin structures, these methods are fundamentally limited. From this method complex magnetization profiles require folding or assembling substructures, which introduce additional post‐processing and restrict the design space, leaving truly arbitrary 3D programming unattainable.

To overcome these limits, more recent strategies employ direct ink writing (DIW) 3D printing, where a strong magnetic field is applied to the uncured elastomer during fabrication, or in‐process. One version of this strategy has magnetic ink inside the printing tip subjected to a strong field via an electromagnet, enabling automatic magnetic alignment along the printing direction [10, 19, 34]. This approach eliminates post‐processing and allows 3D structures to be printed with tailored magnetization. Crucially, however, the magnetization remains confined to the printing path, which means the profile is constrained to the 2D printing plane. As a result, structures requiring magnetization orthogonal to their surfaces—such as arbitrarily shaped thin shells—are difficult to realize, since the printing direction necessarily follows the tangent of the surface.

Subsequent approaches introduced an external magnet capable of moving independently and rotating in 3D to apply impulse fields during printing [35, 36]. Various iterations of this employ either curing immediately after magnetization or melting via a laser and magnetizing in activated spots post‐curing. While this class of approaches advance toward arbitrary 3D magnetization, they remain limited, since the magnet is always positioned beneath the substrate, limiting the largest magnetization to regions near the base. As a result, fabricated designs using free magnets beneath the substrate were still restricted to flat 2D geometries, such as hinges.

**SCHEME 1:**
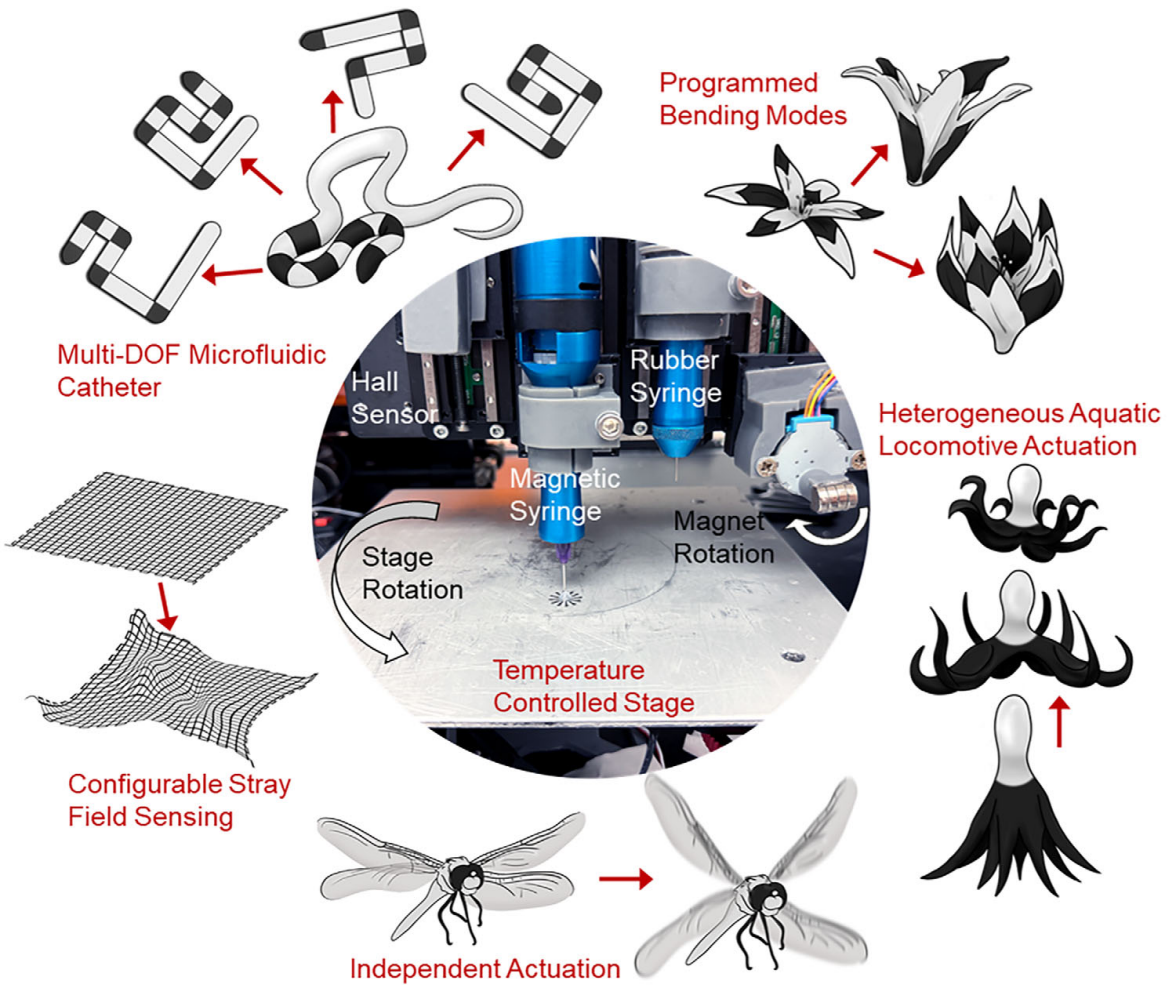
The fabrication system supports customizable multi‐material printing of magnetic and non‐magnetic elastomers while enabling unconstrained, in‐process 3D magnetization. Targeted heating from the print substrate cures each layer in real time, locking magnetic domains into prescribed orientations for programmed actuation. This approach produces diverse functional behaviors, including mesh‐based stray‐field sensing, independently actuating bioinspired wings, heterogeneous aquatic propulsion reminiscent of octopus tentacles, flower‐type folding patterns, and a magnetically guided serpentine catheter capable of negotiating complex pathways.

Importantly, existing strategies are limited in that they either enable fabrication of 3D structures or allow arbitary magnetization, but not both simultaneously in fully three‐dimensional profiles. Here, we describe a method for achieving fully 3D magnetization profiles in printed structures that exhibit localized bending behaviors. An external magnet positioned above the print is translated in three dimensions and rotated about two independent axes, providing control over the magnetic field at arbitrary locations within the structure. This magnetization process is applied after each printed layer, allowing the creation of custom, homogeneous magnetization profiles throughout large 3D architectures. By coupling this in‐process magnetization control with multi‐material printing, we fabricate anisotropic 3D architectures with customizable magnetization spanning micron‐ to millimeter‐scale features (Scheme 1). The versatility of this platform is demonstrated through several representative systems, including a highly deformable magnetic mesh with dual actuation and sensing functionality; a flower that mimics blooming through programmed bending; a dragonfly with oppositely magnetized wings that generate heterogeneous motion; an octopus‐inspired robotic swimmer with heterogeneously magnetized legs,; and a serpentine fluidic catheter capable of high degrees of freedom (DOF), tortuous‐path navigation, and integrated microfluidic operations such as self‐lubrication and drug delivery.

## 2 Results and Discussion

### 2.1 In‐Process Magnetization Strategy for 3D Printing of Heterogeneous Magnetic Profile

We have developed an in‐process magnetization strategy for 3D‐printed neodymium‐iron‐boron (NdFeB)–elastomer composites, aligning magnetic domains before curing to achieve strong, programmable 3D magnetization. By synchronizing curing kinetics with magnetization, low‐viscosity composites enable domains to align efficiently, resulting in higher net magnetic fields and greater bending deformations than post‐cured methods [15, 17, 20, 22, 30, 31, 32, 33]. Optimization of curing, viscosity, and magnet height enabled spatially discrete and continuous magnetization while preventing droplet deformation. This approach allows arbitrarily programmed magnetization of ferromagnetic composites during printing, as compared with other 3D printing strategies (Table S1), providing a general framework for fabricating soft, anisotropic, and bioinspired actuators with region‐specific, tunable magnetic responses.

**FIGURE 1:**
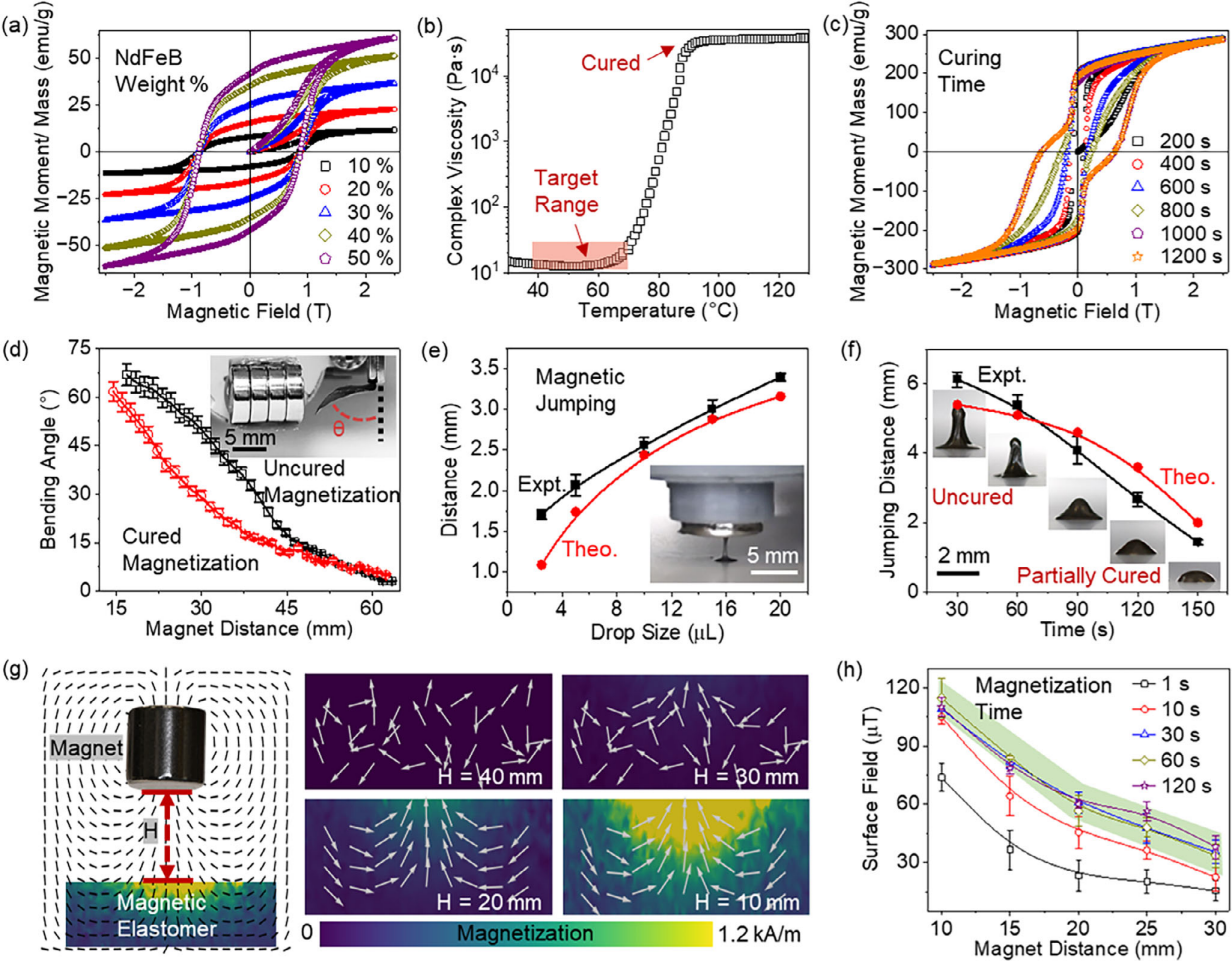
(a) Hysteresis curves for composites with increasing NdFeB weight percentages. The curves maintain similar widths while heights increase, indicating higher saturation magnetization with greater particle loading. (b) Complex viscosity of the elastomer as a function of ramping temperature. Full curing occurs at ∼90°C, so printing is performed at ∼40°C–70°C to allow in‐process magnetization. (c) Hysteresis of a composite curing at 50°C. As viscosity rises, the magnetic susceptibility of NdFeB particles and domain alignment change, widening the curve. (d) Two square composites were magnetized either during curing or after full curing. Bending under an external magnet is greater for the uncured magnetized composite, illustrating the effect of curing on actuation. (e) Jumping distance, defined as the minimum distance at which an uncured droplet deforms toward a magnet, is measured versus droplet volume. Larger droplets increase the effective interaction distance. (f) As curing progresses, the composite stiffens, and the jumping distance decreases, showing reduced deformability. (g) Magnetization of the elastomeric composite by an external magnet at distances of 10–40 mm. Closer magnets induce stronger domain alignment and greater net magnetization. (h) Surface magnetic field measurements as a function of magnet distance and exposure time (1–120 s). Longer exposure and closer proximity yield stronger surface fields, demonstrating programmed, localized magnetization during printing.

The magnetic hysteresis of cured elastomers containing varying weight percentages of NdFeB microparticles was measured to evaluate magnetic coercivity, remanence, and susceptibility (Figure 1a). Across 10–50 wt% loading, the coercivity (width of the hysteresis curve) remained essentially constant, indicating that the intrinsic resistance of the particles to demagnetization is largely unaffected by particle content [11, 37]. In contrast, saturation magnetization and total magnetic moment increased with higher loading, reflecting the greater contribution of magnetic material per unit volume. This trend directly informs the design of 3D‐printed structures: by tuning particle fraction, the magnetic response of printed components can be increased without demanding stronger actuating fields [37].

We aim to magnetize the composites in‐process rather than post‐process, which requires magnetization to occur on a time scale compatible with curing during fabrication. To print and cure the elastomeric composites, we use a heated print bed to initiate cross‐linking. Shorter curing time is governed here by temperature, so we target a range that aligns elastomer curing with the magnetization and printing time, approximately 5–15 min. We first characterize the bed temperature (Figure S1), which we aim to heat and cool on demand during printing, using an infrared (IR) camera to measure temperature ramping, cooling, and dwell behaviors. To further refine in‐process curing, we measure the complex viscosity of an uncured elastomer without magnetic microparticles as a function of temperature (40°C–100°C, Figure 1b). The viscosity exhibits a sigmoidal trend: it remains nearly constant near room temperature (30°C–70°C), increases steadily between 70°C and 90°C, and plateaus upon full curing (>90°C). The optimal temperature range for synchronized in‐process curing and magnetization is 40°C–70°C, where cross‐linking begins, enabling controlled fabrication of magnetizable elastomeric structures.

To better quantify curing behavior, we measured the complex viscosities of the elastomer at different fixed temperatures as a function of curing time (Figure S2a). Across all temperatures, viscosities began near 10 Pa·s, which is readily extruded from a dispensing tip and consistent with reported ranges for DIW of viscous magnetic elastomers [10, 14, 30]. This value increased to a steady‐state value of ∼15 kPa·s upon full curing, with higher temperatures accelerating cross‐linking. Specifically, composites reached full curing at approximately 5 min (70°C), 9 min (60°C), 20 min (50°C), and 40 min (40°C). Based on these results, a bed temperature of 60°C was selected, as the 9 min curing time aligns with the desired time scale for simultaneous in‐process curing and magnetization. To confirm that the rheological behavior is largely unchanged with the addition of magnetic NdFeB microparticles, further viscoelastic testing is performed on the elastomer at various temperatures at a fixed shear rate with 40% magnetic loading by weight (Figure S2b). The trend is similar to that of the non‐magnetic elastomer, with a predicted increase in viscosity and unchanged temperature‐sensitive curing behavior.

With the in‐process curing strategy established, we next measured the compressive and tensile moduli of the magnetic elastomer as a function of NdFeB weight percentage, while also evaluating the effect of heat curing versus ambient curing on material flexibility. Maintaining flexibility is critical for manufacturing highly deformable actuators and sensors. While the specific response to stress depends on material geometry, we study two relevant modes of stress: tension in a long, thin composite, and compression of a large bulk solid. Composites with six different weight percentages were tested under ambient curing at room temperature and heat curing at 50°C (n = 5). Compressive stress‐strain curves and associated Young's moduli are shown in Figure S3. As particle content increased, Young's moduli rose from ∼7.9 to ∼13.0 MPa for ambiently cured composites and from ∼9.5 to ∼13.0 MPa for heat‐cured composites. Tensile moduli (n = 5) were similarly measured for thin composites (Figure S4a–c), increasing from ∼7.9 to ∼12.5 MPa for ambient curing and from ∼8.7 to ∼12.3 MPa for heat curing with increasing NdFeB content. Additionally, to enable comparison with standardized material properties, tensile testing was conducted using dog‐bone‐shaped specimens (60 mm length, 6 mm gauge width, 12 mm grip width, and 3 mm thickness) prepared from the 40 wt% formulation (Figure S4d). In both compressive and tensile tests, the effect of heat curing was most pronounced at lower particle fractions, with a minimal difference (∼9.6%) in modulus at higher loadings. Based on these results, 40 wt% NdFeB was selected to maximize magnetic response while preserving printing resolution of ∼50‐100 µm and material flexibility (∼10 MPa) [38].

To examine the time‐dependent magnetic properties of the composite during curing, the 40 wt% NdFeB elastomer was cured at 50°C while undergoing hysteresis measurements (Figure 1c). The shape of the hysteresis curves evolves significantly over time, reflecting changes in material viscosity during cross‐linking. Measurements were performed under an axial, slowly alternating external magnetic field, which appears to influence domain alignment in ways not central to the present study; for instance, even after full curing, the curves differ from those obtained on fully cured composites, suggesting that the alternating field introduces preferred energetic states in the magnetic domains. More directly relevant to our aims, the hysteresis behavior changes markedly as curing progresses. Early cycles, when the material is less viscous, exhibit larger slopes and greater susceptibility, while subsequent measurements reveal widening hysteresis loops as cross‐linking proceeds. The coercivity as a function of time from these courves is presented in Figure S5. These results indicate that magnetic domains align more readily with the external field in low‐viscosity, uncured composites, suggesting that in‐process magnetization is more efficient than post processing magnetization since the willingness to align with an external field is higher before significant curing occurs [39]. This idea is interesting and a clear strength of the processing strategy, but requires more thorough examination.

To achieve strong magnetization—particularly when using less powerful magnets compatible with 3D printing—it is advantageous to magnetize the composites before full curing. To test this and verify the hysterisis measurements, two sets of thin composites were printed and magnetized under identical conditions, differing only in the curing stage during magnetization. The first set was magnetized immediately after printing, still uncured, while the second set was magnetized 3 h later, after fully cross‐linking and solidification. Stray magnetic fields were measured on the top and bottom surfaces of each composite (Figure S6). The results show that composites magnetized prior to curing exhibit higher net magnetic fields than those magnetized post‐curing. Notably, even for thin composites (thickness ∼200 µm), the surface closest to the external magnet (top) consistently exhibited higher stray fields, highlighting the influence of proximity during in‐process magnetization. This stronger magnetization in uncured composites directly translates into an enhanced mechanical response, as the increased magnetic moment allows the material to bend more effectively under an external field. Thin composites were fixed at a corner and allowed to bend while an external magnet approached at a constant velocity, with bending angles recorded (Figure 1d, full sequence in Movie S1). Remarkably, composites magnetized prior to curing exhibited significantly larger bending angles, demonstrating that in‐process magnetization enhances actuation.

To further evaluate the through‐thickness magnetization behavior observed in Figure S6, cylindrical composites (∼10 mm height, ∼10 mm diameter) were fabricated and magnetized in‐process using either conventional bottom magnetization or top inter‐layer magnetization (Figure S7). Ten samples were printed using identical ink formulations and processing conditions for each configuration, with magnetization applied either from below or from above during fabrication. After curing, the axial magnetic surface fields at both the top and bottom surfaces were measured. For samples magnetized from below, the magnetic field experienced by newly deposited layers progressively decreased with increasing print height due to the rapid spatial decay of the external magnetic field. Consequently, the upper regions of the printed structures exhibited substantially weaker magnetic fields and reduced through‐thickness magnetization. In contrast, top inter‐layer magnetization maintained a nearly constant magnet‐to‐layer distance throughout fabrication, enabling stronger magnetic penetration and enhanced top‐surface magnetic fields throughout the structure. The average top‐surface magnetic field for samples fabricated using top inter‐layer magnetization (Figure S7b, red) was approximately 6.2× greater than that of samples fabricated using conventional bottom magnetization (Figure S7a, red), demonstrating the advantage of maintaining a consistent magnet‐to‐layer distance during fabrication.

However, this approach introduces a unique challenge: the uncured elastomer is low‐viscosity and can deform freely, so bringing an external magnet too close can displace the composite. The challenge of bringing a magnet close to a composite to achieve strong in‐process magnetization is inherently dynamic, as proximity affects both magnetization strength and material stability. The process is a positive feedback loop in which, past a threshold, the magnet being close enough to the uncured composite causes slight deformation toward the magnet, which in turn produces a stronger field, more deformation, and so on until the composite leaps off the substrate toward the magnet. To quantify this, the minimum distance at which droplets of different sizes remain undeformed on the substrate without “jumping” toward the magnet was measured (n = 5, Figure 1e, full sequence in Movie S2). As droplet volume increases (2–20 µL), the tendency to jump rises, requiring the magnet to be positioned further away to avoid deformation. Additionally, as the material cures—either ambiently over hours or via heat in 10–20 min—viscosity increases, reducing jumping propensity. Fully cured droplets remain fixed, while partially cross‐linked composites exhibit intermediate behavior.

To further parameterize this effect, 50 µL droplets were cured at 50°C for increasing durations (30–150 s), and the jumping distance was measured (Figure 1f). As curing progressed, the jumping height decreased sharply, consistent with observed droplet shapes. For example, after 150 s, the jumping height decreased by 76%, from 6.12 mm to 1.43 mm, highlighting that viscosity evolution during curing governs the mechanical stability of uncured composites under external magnetic fields.

These experiments demonstrate strong magnetic effects in the uncured elastomer but do not fully clarify the underlying physical mechanisms. One contributing factor is the magnetic force exerted by the external field on particles, which may either move freely through the fluid, transmit a body force through the matrix, or exhibit a combination of both behaviors. Another effect is the magnetic torque acting on individual particles or aggregates. A complementary contribution arises from magnetization, as domains within the particles reorient in response to the applied field. To better understand how these interconnected effects interact, we develop three theoretical models to quantify bulk fluid jumping, particle‐level migration, and particle‐level rotation. These models incorporate experimentally measured parameters, including magnetic hysteresis, viscosity, and source field profiles, and are fitted to the data (Figure S8).

First, we parameterize a previously observed phenomenon: magnetic jumping of droplets with varying height and volume as a cylindrical magnet approaches from a distance of 15 mm. The increasing magnetic field and gradient induce deformation against gravity (Figure S9). The predicted jump heights show excellent agreement with theoretical values (Figure 1e,f). From these results, deformation begins at a magnet separation of ∼5 mm for all droplet volumes, with larger droplets exhibiting greater deformation. The dependence of jumping on cure time, and thus viscosity, is also modeled and agrees well with experiments, with jumping initiating at ∼6 mm separation.

Next, we model the force acting on magnetic microparticles. A dimensionless parameter, defined as the ratio of magnetic to gravitational forces, is used to determine whether upward migration or downward settling is favored (Figure S10a). The crossover position for our system prior to curing is ∼4.6 mm. Thus, if the fluid were fixed and particles could migrate freely, upward motion would occur when the magnet is closer than this distance. For a migration time of 1 min at fixed viscosity, the predicted migration distance as a function of magnet height is shown in Figure S10b. Before curing, the maximum migration is ∼700 µm, while after 3 min of curing, it is reduced to ∼6 µm. Although particle migration is possible, the bulk jumping regime encompasses this range, indicating that bulk deformation occurs prior to significant single‐particle migration.

Finally, to demonstrate that the net magnetization in the fabricated composites arises from rotational alignment with the external field, we model the magnetic torque on individual particles. The rotation angle is calculated as a function of time for fixed magnet heights and curing times, further supporting this mechanism (Figure S11).

To investigate the magnetization behavior, which theory suggests is dominated by rotational alignment, we simulated how magnet height influences magnetization within the composite (Figure 1g) using a time‐independent finite element analysis (FEA). A full simulation code and methods are shown in Figure S12. Arrows in the simulation illustrate domain alignment as the magnet is lowered from 40 to 10 mm above the composite. When the permanent magnet is far from the composite, magnetic domains remain randomly oriented, resulting in negligible net magnetization. As the magnet approaches, particles rotate at an angular speed determined by the magnet distance, orientation, and material viscosity. Additionally, microscale magnetic domains within the particles or clusters reorient according to the measured hysteresis behavior. The combined contributions of particle rotation and domain reorientation produce a net magnetization that can be parameterized and programmed.

We next sought to experimentally parameterize both magnet height and total magnetization time. Multiple small droplets (50 µL, n = 5) were extruded and magnetized at varying heights and durations. The resulting surface magnetic fields were measured and are presented as five curves for different magnetization times in Figure 1h. As expected, bringing the magnet closer to the composites (down to 10 mm) and holding it in place for longer (up to 2 min) increased the magnetization. Importantly, magnetization saturates for the specific external field applied: there is little difference between 10 and 120 s of exposure (surface fields of ∼105 and ∼110 mT, respectively), indicating that short magnetization times are sufficient. By contrast, magnet height strongly affects the resulting magnetization, with surface fields ranging from ∼105 to ∼64 mT at 10 and 15 mm after just 10 s, highlighting the need for careful height optimization to maximize actuation. FEA simulations further confirm these trends, showing increased surface fields with decreasing magnet height (Figure S13). As expected, decreasing the magnet height during magnetization increases the surface field. However, the overall trend shape remains unchanged, with measured and simulated values reaching similar levels (110 µT for 120 s and 121 µT for indefinite magnetization).

To experimentally confirm that magnetization occurs within a regime dominated by bulk jumping and particle migration—which we avoid—and instead enables undeformed domain reorientation and whole‐particle alignment under an external field, we perform X‐ray fluorescence (XRF) measurements (Figure S14). Composites magnetized at different heights and durations are shown in Figure 1f to exhibit significantly different remanent magnetizations, which we attribute to rotational alignment.

To further confirm that this effect is primarily rotational rather than migrational, as predicted by our theoretical model, we subject composites to variable magnetic field conditions, then bisect the samples and measure the elemental composition of the top and bottom halves. A measurable difference in composition is observed as a function of magnet height. In general, the top half is less rich in iron and neodymium than the bottom half. For example, in the control sample without magnetization, the iron content differs between the top and bottom regions. Across most magnetization conditions, the bottom half remains richer in iron and neodymium; however, at a magnet height of 5 mm, the compositions are nearly uniform (∼34.55% and ∼33.69% iron in the top and bottom halves, respectively). The increased iron content in the bottom region for weaker magnetization conditions is consistent with particle settling, as predicted by our model.

To evaluate particle redistribution within the elastomeric matrix during magnetization and curing, two samples were prepared under identical conditions using magnet heights of ∼10 mm and ∼0 mm, respectively (Figure S15). Samples were fabricated in molds rather than by printing to isolate magnetization‐induced effects and minimize deformation associated with bulk motion. Three‐dimensional micro‐CT imaging was performed to spatially resolve particle distribution, clustering behavior, and internal concentration gradients throughout the sample volume.

The sample magnetized at ∼10 mm maintained a comparatively homogeneous particle distribution with minimal vertical migration (Figure S15a–c). In contrast, the sample magnetized at ∼0 mm exhibited noticeable vertical particle redistribution, where larger particles preferentially accumulated near the upper surface under strong magnetic field gradients (Figure S15d–f). Moderate clustering and local chaining were observed in both samples; however, substantial redistribution occurred only at extremely small magnet distances associated with strong field gradients.

Because the NdFeB particles were not pre‐magnetized prior to mixing, the applied magnetic field first induces magnetic domain alignment within the particles, thereby generating magnetic moments that subsequently experience rotational torque within the uncured elastomeric matrix. Under the optimized magnetization conditions used in this study, micro‐CT analysis indicates that large‐scale translational particle redistribution is minimal, while rotational alignment of magnetized individual particles and small particle aggregates remains the dominant mechanism governing programmable remanent magnetization. While moderate aggregation and local chaining are observed, these effects appear to assist rotational alignment rather than dominate the magnetization process.

As demonstrated previously in Figure 1, the remanent magnetic fields vary significantly as a function of magnet height (Figure 1e–h). Taken together with the theoretical analysis, these observations suggest that stronger magnetic fields enhance particle magnetization and rotational alignment, whereas excessively small magnet separations generate strong field gradients capable of inducing bulk fluid displacement and particle/aggregate migration. Therefore, the printing process was parameterized to achieve strong remanent magnetization while avoiding significant deformation or particle redistribution. Having optimized these conditions, we proceeded to magnetize thin elastomeric solids in discrete regions to achieve spatially programmed actuation.

### 2.2 Discretely Magnetized Actuating Structures

**FIGURE 2:**
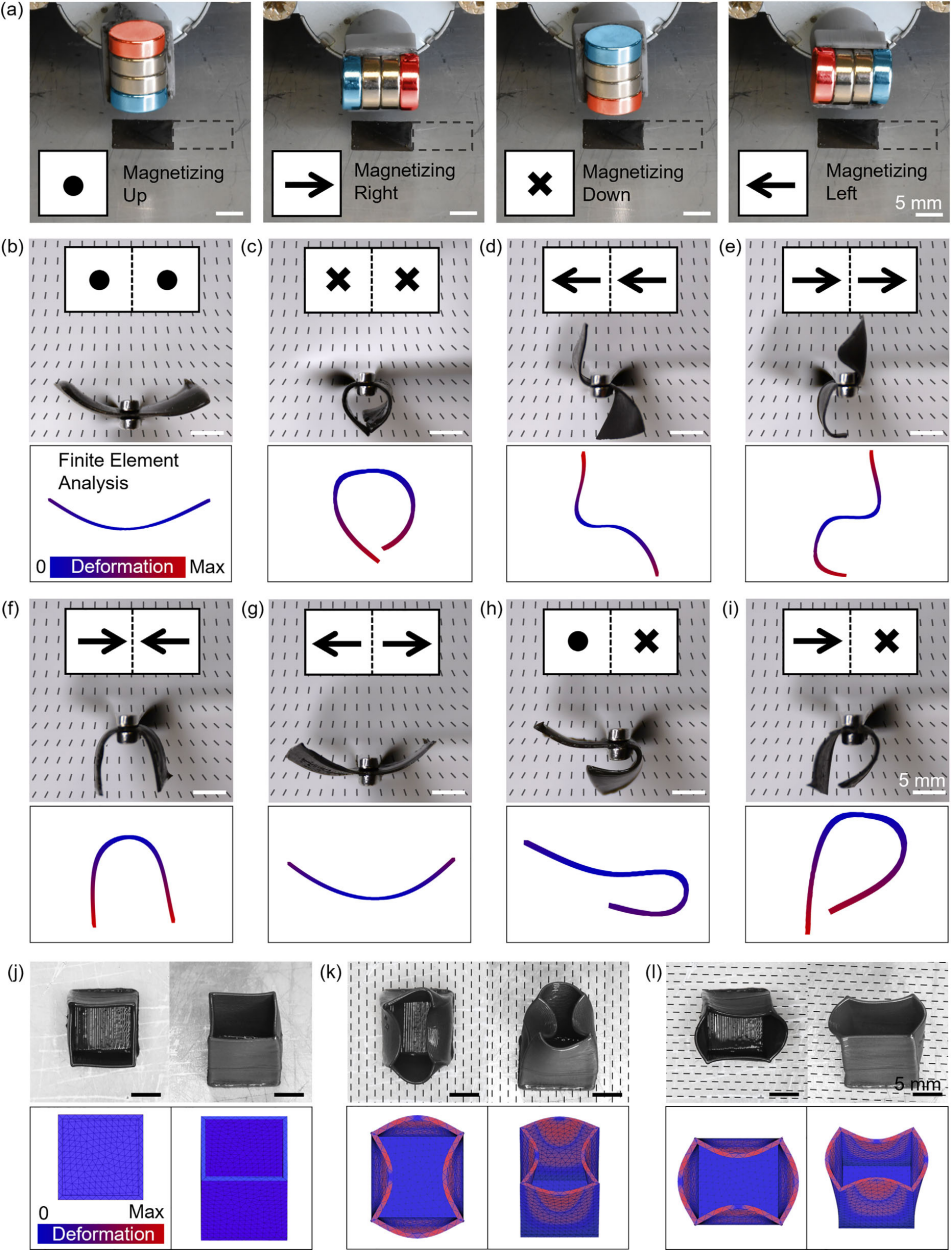
Discrete 3D magnetization of thin composites (200 µm in thickness, 5 mm in width, and 4 cm in length) for programmed bending. (a) Schematic of the top view of composites, each magnetized in two regions with one of four directions: up, right, down, or left. (b–e) Four thin rectangular strips are printed and uniformly magnetized in a single direction (up, down, left, or right), fixed at their center, and allowed to bend under an external magnet above. Each strip exhibits a distinct bending pattern, consistent with FEA predictions. (f–i) Four additional strips are magnetized in two discrete halves in differing directions and subjected to the same external field. The resulting bending patterns reflect the discrete magnetization regions and agree with FEA simulations of magnetic mechanics. (j–l) A thin cubic shell is 3D‐magnetized with each face oriented normal to its surface, creating four independent magnetic moments. Under external fields applied along the (k) −*Y* and (l) −*X* axes, the shell undergoes competing wall deformations that closely match FEA predictions.

To explore programmable actuation, we first designed discretely magnetized structures that enabled the investigation of non‐uniform magnetic profiles. A top‐down schematic of the rectangular strips indicates magnetization directions: ʻ  ·  ʼ indicating magnetization out of the plane, ʻ × ʼ indicating magnetization into the plane, and ʻ ← ʼ and ʻ→ʼindicating magnetization right to left or left to right (Figure 2a). Each strip was printed and magnetized in two distinct phases/regions (left and right sides), forming eight discrete rectangular strips: ·   ·, × ×, ← ←, →→, →←, ←→, ←→, ·   ×, →×.

Upon printing and magnetization, the strips were fixed about the center by a sample holder, allowing them to bend under a uniform external magnetic field applied from above. Using FEA with the tensile modulus of the magnetic elastomer, magnetization parameters informed by hysteresis data, and a uniform external field, the bending profiles of the strips are shown below each experimental result (Figure 2b–i).

Under the influence of gravity, the composites all preferentially bend downwards more easily than they bend upwards. As both sides of the strips are uniformly magnetized, a single direction exhibits markedly different bending behaviors (Figure 2b–e). The magnetization profiles along the positive *Z*‐axis (+*Z*) (Figure 2b) or negative *Z*‐axis (–*Z*) (Figure 2c) tend to curl up to maximize the attraction between the uniformly magnetized faces and the external field. In contrast, the orientations along the *X*‐axis (Figure 2d,e), bend enough to align the surface with the external field, but not further. Strips bending with or against gravity display distinct shapes, highlighting the interplay between magnetization orientation, gravity, and the external field in programming complex actuation.

As strips were magnetized with two distinct phases on the left and right sides (Figure 2f–i), these non‐uniform magnetization profiles produced four unique bending shapes compared to those of the uniform magnetization profiles. We move to print, discretely magnetize, and actuate a 3D structure rather than 2D strips. The simplest 3D structure, which is thin enough to demonstrate high flexibility and magnetic compliance, is a cubic shell, magnetized in 3D with a symmetric magnetization direction chosen to be pointing normal to each cube wall, creating four independent magnetic moments pointing out of the cubic shell (Figure 2j, full sequence in Movie S3). The cubic shells are subjected to four competing forces from the cube walls from external magnetic fields traveling top to bottom along the −*Y* axis (Figure 2k) and left to right along the −*X* axis (Figure 2l), and are compared to simulated results of the cube subjected to identical purely mechanical forces via FEA.

These results indicate that bending arises from the interplay of magnetization direction, alignment fraction, external field dynamics, and material mechanics: magnetic forces generate the driving torque, while the material's mechanical properties determine how that torque translates into physical deformation, and partial domain alignment combined with the external field introduces asymmetry, enabling diverse, programmable shapes. In thin composites, this interplay is particularly pronounced: attraction often dominates, causing twisting toward attractive interactions, while partial saturation (fields < 1 T) creates asymmetric attraction and repulsion as randomly aligned domains compete with domains oriented opposite the external field. The mechanical compliance of the material further amplifies these effects, resulting in complex, controllable bending behaviors.

A butterfly‐shaped composite demonstrates this asymmetry (Figure S16). The entire structure is uniformly magnetized in the positive *Z* (*+Z*) direction, normal to the surface, with identical magnetic volumes on both sides of the hinge. If only domain‐generated forces acted, both sides would bend symmetrically: attraction in one orientation and repulsion in the other. In practice, when the external field is attractive, both sides bend upward. When the field is reversed, the halves behave differently: the right side, with magnetic volume near the hinge, experiences repulsion, while the left side, with volume farther from the hinge, twists upward, mechanically favoring the attractive bottom face.

This complex interaction of magnetic and mechanical effects is critical for achieving programmable bending. These simple constrained strips demonstrate multiple actuation modes, providing a foundation for larger, dynamic 3D structures capable of more sophisticated shape transformations.

### 2.3 Discretely Magnetized Sensors

**FIGURE 3:**
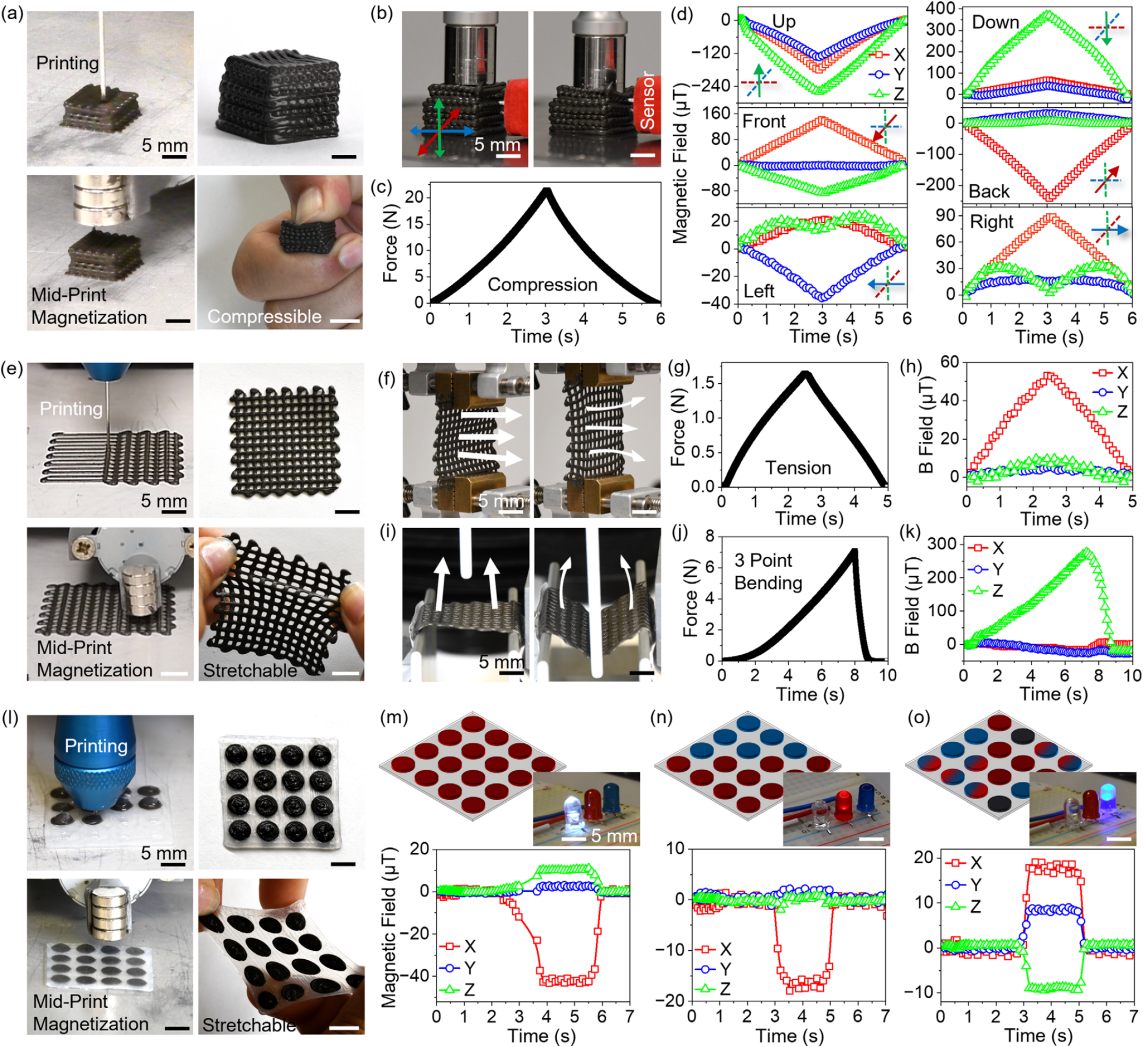
Sensing behavior of discretely magnetized solid composites. (a) A cubic sensor is printed and magnetized in‐process, then compressed to measure changes in the stray magnetic field. (b–c) Six magnetization states are subjected to compression, producing measurable bending patterns. (d) The resulting stray field changes are detected using a Hall sensor. (e) A flexible mesh is printed and magnetized in‐process, then (f) subjected to tensile stretching. (g–h) The tensile deformation alters the stray field, which is captured by a Hall sensor. (i) The mesh is further tested under three‐point bending, (j) measured mechanically, and (k) the induced stray field change is recorded with a Hall sensor. (l) A multi‐material flexible keycard is printed with sixteen discrete magnetic pixels, each magnetized in a unique direction to create distinct stray field profiles. (m) Key 1, magnetized uniformly, produces a strong *x*‐component peak when inserted into the reader. (n) Key 2, with six pixels up and ten down, produces a weaker *x*‐component signal. (o) Key 3, magnetized arbitrarily in up, down, left, right, or unmagnetized, generates *X*, *Y*, and *Z* component signals. Each key produces a unique Hall sensor reading, which triggers a corresponding LED pattern via Arduino programming.

Printing flexible solids with programmable magnetization profiles provides opportunities not only for magnetic actuation but also for magnetic sensing [26, 27, 28, 29, 40, 41, 42, 43], changes in magnetization can be directly mapped quantitatively onto mechanical deformation. While specific device configurations may vary, the underlying sensing principles are similar. Flexible elastomers interact with a programmed magnetic field, either generated by an attached permanent magnet or encoded within the material during fabrication. As the elastomer deforms (e.g., under compression), the surrounding magnetic field distribution changes. 3D Hall sensor, capable of detecting stray fields in the micro‐Tesla range, enables precise quantification of stretching and compression in such solids [44]. In order to test this concept, we printed a cubic solid and magnetized it in situ with the magnetization direction set normal to the substrate (Figure 3a). We print a bulk solid here rather than a shell, since the volume fraction increases the total stray field. Notably, other magnetization methods in‐process fail to print this simple cube because it is non‐planar and has a uniform magnetization directed normal to the printing plane. As shown earlier (Figure S6), magnetization depends strongly on spatial position: even for a thin 200 µm strip, the face closer to the magnet is magnetized more strongly than the opposite face. If a large cube (∼1 cm^3^) is magnetized only after printing, the result is weak and non‐uniform. To overcome this, we applied magnetization intermittently between printed layers, which produced a strong and uniform out‐of‐plane magnetization. We then explored six principal magnetization orientations while the cube was mechanically compressed (Figure 3b,c). During uniform compression, the cube's stray magnetic field—measured without any external driving field—changed in a detectable way.

In Figure 3d, 3D Hall sensing results are shown for cubes magnetized in six orientations: ‘up,’ ‘down,’ ‘front,’ ‘back,’ ‘left,’ and ‘right,’ corresponding to +*Z*, −*Z*, +*Y*, −*Y*, +*X*, and −*X* directions. Each orientation produces a distinct response curve as the *X*, *Y*, and *Z* components of the stray magnetic field deflect and recover under compression. For example, flipping the magnetization from up to down reverses the *Z*‐field response from a decreasing to an increasing trend. Similarly, changing from front to back inverts the *X*‐field response, while switching from left to right shifts the *y*‐field from a slight decrease to a slight increase. These behaviors illustrate characteristic trends in the sensing response, though the patterns are not fixed or immutable.

The orientation of the Hall sensor—i.e., the choice of its *X*, *Y*, and *Z* axes—is arbitrary, and its position relative to the magnetic solid is equally arbitrary, meaning that even small changes in placement can significantly alter the measured response. Because the magnetic field is spatially dependent, the Hall sensor effectively acts as a point probe, capturing the local field at a single position as it evolves over time and under applied strain. As a result, the absolute sensor values and response trends are not fixed. Nevertheless, when the sensor is kept in a fixed position, reproducible and characteristic patterns emerge. For instance, the ‘right’ and ‘left’ magnetized cubes consistently produce an *M*‐shaped response curve along the *Z* axis. This effect arises because during compression the volumetric center of the cube shifts relative to the sensor, altering the detected field. Compressive Hall sensing and mechanical testing of the magnetized cube were performed over 2000 cycles (Figure S17). Both the cube's mechanical response and the corresponding stray magnetic field measurements remained highly stable throughout, demonstrating excellent repeatability.

To demonstrate the same sensing principle under tension, we printed a highly flexible, stretchable mesh and magnetized it in‐process, with the magnetization direction oriented normal to the substrate (Figure 3e; Movie S4). The mesh was first subjected to tensile stretching (Figure 3f; Movie S5), and the corresponding stray field deflection showed an increase in the *X*‐component followed by restoration (Figure 3g,h). The peak stray field is smaller than that of the cubic sensor due to the mesh's smaller magnetized volume. The mesh was similarly cycled through 2000 stretching cycles (Figure S18), during which both its mechanical response and stray‐field measurements remained highly stable, demonstrating excellent repeatability. Next, the mesh sensor was tested under a different deformation mode: three‐point bending (Figure 3i). This bending produced a distinct stray field deflection (Figure 3j,k), with a peak in the *Z*‐component followed by restoration.

Notably, for all strain measurements—cubic compression, mesh tension, and mesh bending—the mechanical deformation and Hall sensor measurements occurred on identical time scales. These results demonstrate that the sensing is immediate, well‐defined, and effective even at a distance.

The final application demonstrates magnetic stray field sensing without relying on flexure or magnetoelastic effects. We fabricated a flexible, multi‐material ‘keycard’ containing sixteen small circular magnetized ‘pixels,’ which could be applied to door locks, keypads, or keyboards [45]. The base structure, made of pure silicone rubber, was printed first, followed by embedded printing of the sixteen circular magnetic pixels from a magnetic elastomer and magnetized in custom orientations (Figure 3l). The resulting structure is thin (∼300 µm), flexible, and precisely programmed in its magnetization profile. Three keycard designs of increasing complexity were produced. Each keycard was inserted into a single slot connected to a Hall sensor, which was programmed to respond to specific 3D stray‐field patterns generated by the magnetization profile (Figure S19 shows the Hall sensor setup, and Figure S20 illustrates the Hall sensor LED coding, Supporting Information).

First, a keycard was printed and magnetized uniformly, with all sixteen pixels oriented ‘up’ (red, Figure 3m). When inserted into a slot, the Hall sensor reads a strong, distinct peak in the −*X* direction. Next, a keycard with six pixels magnetized ‘down’ (blue) and ten pixels ‘up’ (red, Figure 3n) produces a different −*X* signal of weaker magnitude. Finally, a third keycard was printed with arbitrarily programmed magnetization in either no direction (black) or one of four principal directions (red = ‘up,’ blue = ‘down,’ red‐to‐blue pattern = ‘right‐to‐left’ or ‘left‐to‐right,’ Figure 3o), generating a unique, measurable stray field with components in +*X*, +*Y*, and −*Z*. Each keycard is programmed to trigger a specific combination of LEDs when inserted, producing a distinct light pattern based on its magnetic profile, as read by the Hall sensor (Movie S6).

The keycard demonstration illustrates the versatility of spatially programmable magnetization for discrete, contactless sensing applications. By selectively magnetizing individual pixels in custom orientations, each keycard generates a 3D stray field pattern that a Hall sensor can reliably read. This approach allows multiple keycards to be distinguished based on their magnetic signature alone, without requiring mechanical deformation or electrical circuitry within the cards. The magnitude and direction of the sensed field can be tuned by adjusting the number, orientation, and spatial arrangement of magnetized pixels, enabling scalable encoding for secure access or input devices [46].

### 2.4 Continuously Magnetized Actuating Structures

**FIGURE 4:**
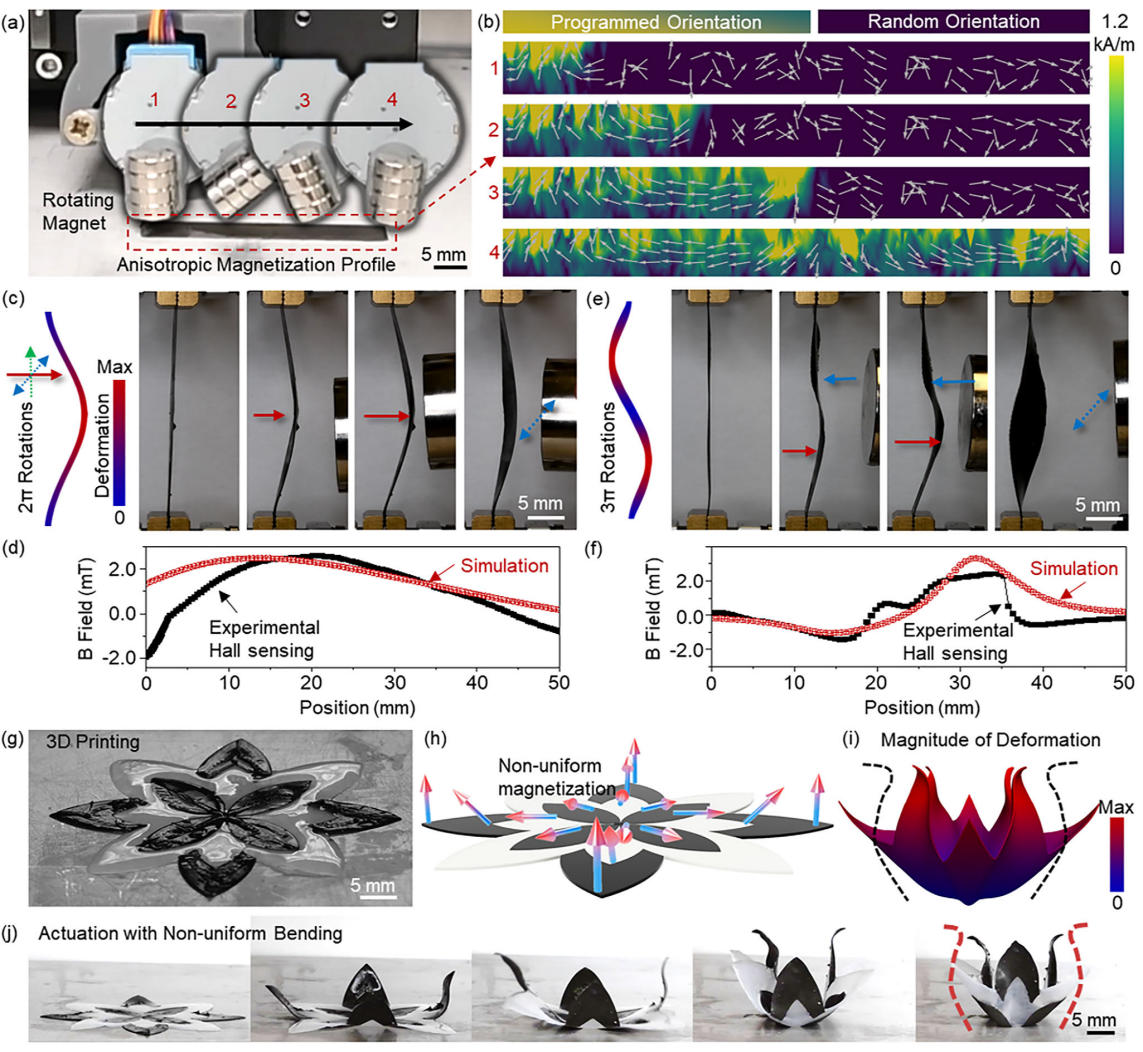
Continuous magnetization of thin composites for programmed bending. (a) A long strip is magnetized in‐process with a rotating external magnet. As the magnet moves along the strip, it rotates, imparting a continuous, anisotropic magnetization profile. (b) Simulation of the strip under the rotating magnet demonstrates the formation of a continuous, spatially varying magnetization profile. For a 2π rotation along the strip, (c) experimental and simulated bending FEA, and (d) stray field measurement agree and impart a unique bending shape, producing a distinct bending shape characterized by a single peak at the strip center (*L*/2) that is attracted to an external magnet. In case of a 3π rotation, (e) the experimental and simulated bending (f) together with stray‐field measurements again align, yielding a more complex deformation profile with simultaneous attraction and repulsion occurring at *L*/3 and 2*L*/3. (g) Applying the same continuous magnetization principle to a multi‐material flower, petals are magnetized in‐process during printing. (h) The resulting non‐uniform magnetization creates a bending pattern unattainable with uniform magnetization, (i) demonstrated via FEA, and (j) experimentally, as the flower folds upward under an external magnetic field.

In contrast to discretely magnetized regions, we investigate solids with fully continuous, non‐uniform magnetization profiles achieved through in‐process magnetization. Complex 3D bending behavior requires correspondingly intricate magnetization patterns, making careful parameterization essential. To illustrate this concept in a 1D example, we study a long, thin strip subjected to a varying magnetic field from an external rotating magnet oriented in the *X*–*Z* plane (Figure 4a, full sequence in Movie S7). The magnet moves along the *X*‐axis via a Gantry motion controller. At the same time, the magnet's position, angle, speed, and angular velocity are precisely controlled so that it completes a predetermined number of rotations along the strip. This motion is repeated as the strip cures, allowing the continuous magnetization pattern to solidify according to the programmed design. As the cure proceeds, viscosity rises, and the same particles transition from mobile to effectively frozen, preserving whatever partial orientation distribution exists at that time.

This principle of continuous magnetization rotation is illustrated in a time‐dependent FEA simulation (Figure 4b). These simulations were performed as quasi‐static, field‐driven deformations in which the programmed magnetization profile interacts with a spatially varying external magnetic field to generate bending‐dominated actuation. The simulations are intended to provide physically informed, qualitative predictions of deformation behavior and demonstrate how actuation direction and mode can be reproducibly programmed through spatial control of magnetization. As the external magnet passes over the strip, it generates a dynamic magnetic field along its length. By fixing the magnet speed, height, and rotational rate, and discretizing the strip into elements, we integrate the magnetic history of each element to determine the spatial distribution of magnetization strength and direction. Once magnetized, these regions retain a remanent field, which remains stable as the magnet moves over other areas, and as the viscosity increases during curing. At a fixed magnet height of 10 mm, the micro‐scale magnetic domains gradually align with the changing external field, producing a continuous, non‐uniform magnetization profile along the *X*‐axis. Regions with strongly aligned magnetization are colored yellow, while areas with random or negligible magnetization are colored dark blue. Magnetization is stronger near the strip surface, as it is closer to the external magnet. This effect was also observed experimentally (Figure S6), where the surface nearest the magnet exhibited a noticeably stronger stray field compared to the opposite surface, even in thin samples.

To experimentally validate this magnetization, compare the measured magnetization profile with finite element analysis, and analyze the resulting bending behavior, we fabricated and magnetized two strips at the same magnet height of 10 mm for 20 cycles over 5 min. The first strip was magnetized with a single full rotation of the magnet (2π radians). Ideally, the magnetization profile should point along +*Z* at *L* = 0, −*Z* at *L* = *L*/2, and +*Z* at *L* = *L*. Both ends of the strip were fixed, and under the influence of an external magnet, the center of the strip exhibited an attractive bending consistent with the expected magnetization pattern (Figure 4c). While this bending qualitatively matches predictions, it does not quantitatively confirm the intended magnetization profile. To verify this, a Hall sensor mounted on the Gantry motion stage was scanned over the strip to measure the *Z*‐component of the magnetic field along its length (Figure 4d). These experimental measurements (black) closely match the *Z*‐field profile obtained from the FEA simulation of the theoretical magnetization (red), confirming the intended pattern and resulting bending behavior. Although this produces a single central bulge, achieving more complex bending requires introducing additional rotations and variations in the magnetization profile, which we explore in the following experiments.

A second strip, printed identically to the first, was magnetized with 1.5 total rotations (3π radians) along its length. Ideally, this would produce a magnetization profile of +*Z* at *L* = 0, −*Z* at *L* = *L*/3, +*Z* at *L* = 2*L*/3, and −*Z* at *L* = *L*. Mechanical constraints, however, limit the strip's ability to bend perfectly in both directions simultaneously. As shown in Figure S16, attractive bending dominates in regions where attraction and repulsion compete, particularly in solids that have not reached saturation magnetization. Despite these limitations, under the influence of an external magnet, clear regions of simultaneous attraction and repulsion are observed at *L* = *L*/3 and *L* = 2*L*/3 (Figure 4e). To confirm this behavior, the *Z*‐component of the magnetic field was measured with a Hall sensor and compared with the theoretical profile extracted from simulation (Figure 4f). The close agreement between the experimental (black) and simulated (red) profiles demonstrates that continuous in‐process magnetization via controlled rotation produces pronounced and predictable magnetization patterns, enabling precise control over complex bending. We simulate additional strips magnetized by the same method of allowing an external magnet to rotate *N* times over the length of the strip (Figure S21). These results establish that spatially programmed magnetization can be used to design soft actuators with tunable, sophisticated motion, laying the groundwork for applications in soft robotics, adaptive structures, and programmable shape‐morphing devices.

We next explore a more complex magnetization profile in a 2D structure, magnetized in 3D to achieve programmed, intricate bending—a flower folding upward. A planar flower is printed with both magnetic and non‐magnetic petals (Figure 4g), consisting of eight petals: four with magnetic tips and four with non‐magnetic tips. In‐process magnetization is applied after printing with radial symmetry, so that each magnetic petal receives an identical quarter rotation (π/2 radians) from the flower center to the distal tip (Figure 4h). FEA simulates the resulting bending magnitude, which is color‐coded to illustrate petal deformation (Figure 4i). A time‐lapse over 5 s shows the flower folding upward under an external magnetic field (Figure 4j, full sequence in Movie S8). The non‐uniform bending pattern, with *S*‐shaped petals, arises from the programmed magnetic domains aligning with the external field. This type of complex, pre‐programmed folding is highly desirable for precise engineering and actuation applications, motivating the design of three additional cases with specialized magnetization and actuation profiles [4, 11].

These examples illustrate how continuous, spatially programmed magnetization enables precise control over complex 3D actuation/bending behaviors in both 1D and 2D geometries. This versatility establishes a robust platform for designing soft, programmable actuators capable of sophisticated motion, with potential applications in soft robotics, adaptive structures, shape‐morphing devices, and other engineering systems requiring tunable mechanical responses.

### 2.5 Continuously Magnetized Biologically Inspired Actuators

Building on the flower demonstration, we next designed biologically inspired structures with specialized, heterogeneous magnetization profiles to achieve complex, targeted actuation. Each design leverages spatially programmed magnetization to control both the magnitude and direction of bending throughout the structure. The first design focuses on a heterogeneously magnetized dragonfly, where opposing magnetization in the wings produces asymmetric, coordinated motion under a uniform magnetic field. The second design explores an aquatic locomoting robotic octopus, in which each leg is magnetized differently and combined with a non‐magnetic central chamber to balance buoyant forces and enable precise, coordinated swimming‐like motion. The third example is a serpentine catheter with discrete hollow magnetized nodes for high‐degree‐of‐freedom motion, self‐lubrication, and microfluidic delivery. These examples demonstrate the versatility of continuous 3D magnetization in engineering soft, programmable actuators capable of sophisticated, multi‐axis motion that mimics biological behavior.

#### 2.5.1 Heterogeneously Magnetized Dragonfly

**FIGURE 5:**
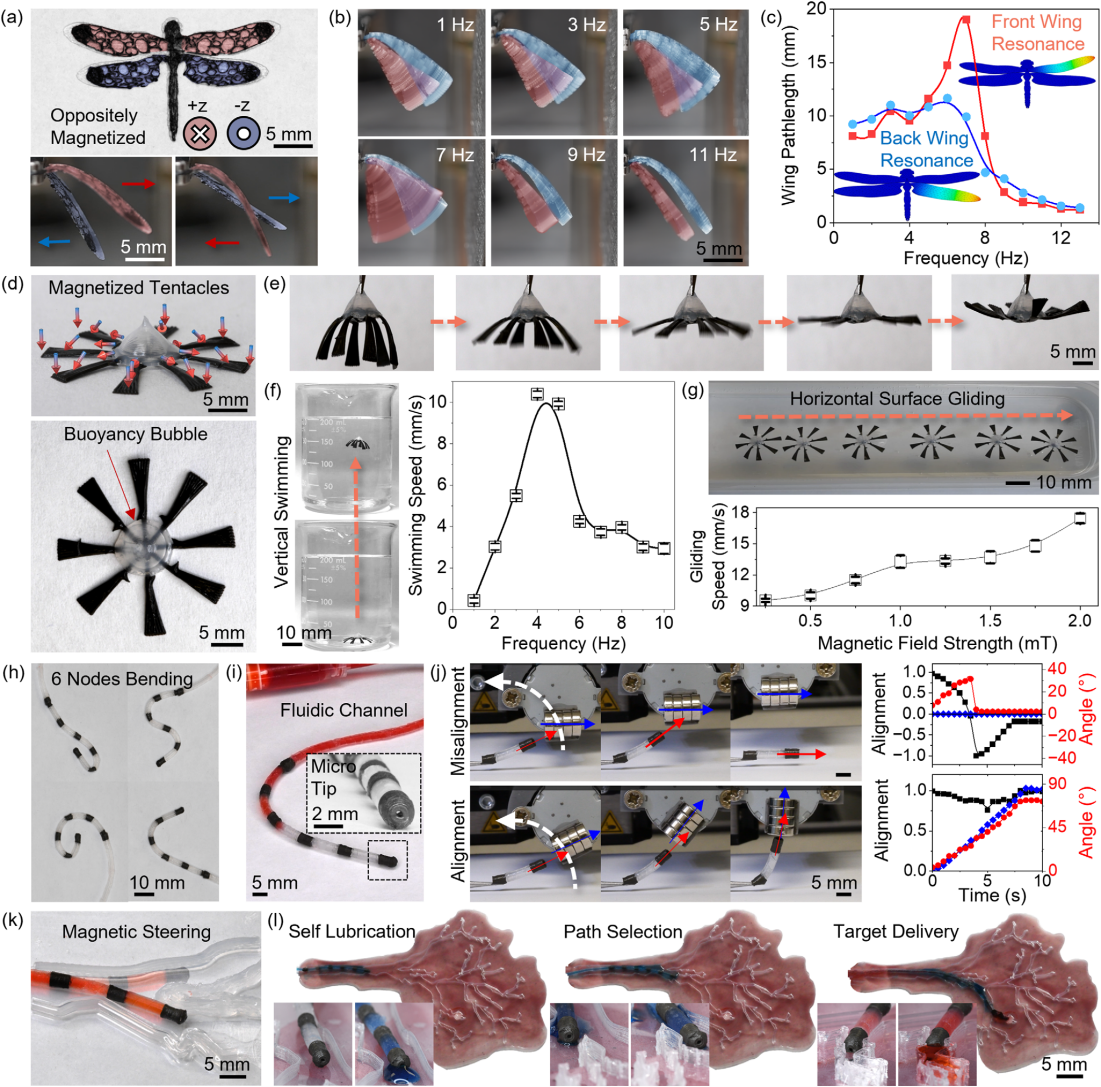
Anisotropic actuation of biologically inspired robotic structures. (a) A dragonfly printed with non‐magnetic elastomer and patterned with magnetic elastomer is magnetized such that the top wings are out of plane (red) and the bottom wings into the plane (blue) for visual clarity. The wings bend in opposite directions under an external magnetic field. (b) An alternating magnetic field generated by an electromagnet produces flapping motion, visualized by overlaying frames from video recordings at different driving frequencies. Maximum wing path length occurs near the resonant frequency of ∼ 7 Hz; at higher frequencies (e.g., ∼ 11 Hz), wing motion is limited. (c) Wing path length as a function of driving frequency confirms resonance (∼ 7 Hz), with FEA predicting resonant modes of ∼ 6.83 Hz and ∼ 6.32 Hz for front and back wings, respectively. (d) A robotic octopus is printed with eight magnetized tentacles for vertical aquatic locomotion. The body is a hollow cone containing a trapped air bubble to maintain neutral buoyancy. (e‐h) Tentacles are magnetized continuously to produce a programmed flapping pattern under an alternating magnetic field, resulting in vertical swimming (e) and a measurable vertical swimming speed as a function of driving frequency, with resonance at ∼4 Hz (f). (g) The flat‐printed bottom enables efficient surface gliding via surface tension, with lateral speed controlled by the strength of a uniform magnetic field, enabling precise 2D navigation on the water surface, while vertical motion is controlled by arm actuation. (h) A hollow serpentine catheter is fabricated with 6 magnetic nodes, allowing high DOF and bending into desired shapes. (i) The catheter is hollow, printed in 3D with a microscale fluidic tip for microfluidic application. (j) A single segment of a free node and fixed node is allowed to bend under the influence of an external magnet, which either rotates to promote high alignment or does not rotate and causes the segment to fall under gravity. Black, red, and blue in the plots correspond to the calculated alignments, the measured angle of the node, and the measured angle of the magnet from the surface, respectively. This device is allowed to steer (k) through a bifurcated environment (l) which mimics real tissular space as the catheter self‐lubricates, steers, and then delivers a payload of red dye to the target, mimicking drug delivery.

To mimic the complex, asymmetric locomotion of a dragonfly with independently actuating wings, anisotropic magnetization is required [47]. A 2 cm dragonfly model is printed using silicone elastomer, followed by embedded printing of patterned magnetic elastomer along the body and wings. Small magnetic elements are patterned at the distal ends of the wings to mimic the pterostigma, which in dragonflies regulates flight dynamics [47]. The top two wings (red) are magnetized out of the plane, while the bottom two wings (blue) are magnetized into the plane (Figure 5a). The red and blue coloration is added artificially for clarity. This anisotropic magnetization profile produces opposing actuation responses under an external field: when the field points to the right, the longer top wings (red) experience repulsion while the shorter bottom wings (blue) experience attraction; when the field points to the left, the top wings experience attraction and the bottom wings experience repulsion. The static mechanics of the wings—constrained at the body and free to bend under magnetic forces and gravity—are simulated (Figure S22). The simulations closely match experimental observations, with the highest stresses occurring near the proximal ends of the wings, close to the body.

The dynamics of the wings are investigated under an alternating magnetic field generated by an electromagnet with variable frequency. The wing arc lengths over their full range of motion are measured for driving frequencies from 1–12 Hz (Figure 5b, full sequence in Movie S9). A driving frequency of 7 Hz corresponds to the resonance of both wing shapes constrained at the body, producing the largest bending angles and arc lengths compared to other frequencies. The arc length as a function of driving frequency for the two wings is presented in Figure 5c. While the anisotropic magnetization successfully emulates the alternating wing behavior of a dragonfly, true locomotive flight involves extremely thin and high‐speed wings capable of actuating dozens to hundreds of times per second, which the wings cannot reasonably reach with 7 Hz resonance. For this study, we focus instead on aquatic locomotion as a more accessible and relevant application of heterogeneously magnetized soft actuators.

The heterogeneously magnetized dragonfly demonstrates how spatially programmed anisotropic magnetization can produce opposing, coordinated actuation across multiple appendages. By independently controlling the magnetization of the top and bottom wings, we achieve distinct bending responses under the same external field, emulating the alternating wing motion characteristic of dragonfly flight. This approach provides a foundation for designing biomimetic actuators for applications that require precise, coordinated movement of multiple elements, such as micro‐robotics, soft grippers, or reconfigurable structures [4, 11, 48].

#### 2.5.2 Aquatic Locomoting Robotic Octopus

With careful design and magnetization, we fabricated a robotic octopus with eight magnetized legs capable of controlled 3D motion. A key design principle is that the non‐magnetic body remains passive, while the magnetized arms provide all actuation. Analogous to a real octopus, which moves its body primarily through coordinated arm movements, the robot achieves locomotion by displacing fluid with its eight limbs [49]. The octopus is carefully engineered to be neutrally buoyant, enabling efficient vertical motion.

Each of the eight arms is magnetized with radial symmetry so that under a vertical alternating magnetic field, the arms bend upward and extend downward in coordinated motion, mimicking natural octopus behavior (Figure 5d). The non‐magnetic elastomeric body is printed as a hollow cone, designed to trap air and generate upward buoyant force. This strategy, inspired by aquatic organisms that control motion through air intake and release, enables the robotic octopus to achieve stable, controllable swimming‐like locomotion using only its magnetized parts [50].

The bending behavior of the eight arms under an external magnetic field is shown in Figure 5e (full sequence in Movie S10). As designed, the arms extend downward due to a combination of gravity and the applied field, then curl upward to displace water and generate locomotion. Similar to the dragonfly wings, the actuation pattern depends strongly on the driving frequency. Increasing the number of full actuation cycles per second enhances net displacement, but exceeding the mechanical resonance frequency results in more frequent, yet incomplete, motions (Figure S23).

To test locomotion, the octopus was placed in a water‐filled beaker within a solenoid producing a 1–10 mT alternating magnetic field of variable frequency. Even with this relatively weak field, the octopus can be remotely controlled to swim upward at variable speeds depending on the driving frequency (Figure 5f). The maximum (resonance) swimming speed occurs near 4 Hz, reaching ∼10.4 mm/s. In a second mode of motion, the flat‐printed bottom of the octopus allows it to float efficiently on the water surface via surface tension. This enables lateral movement across the water independent of arm actuation (Figure 5g, full sequence in Movie S11). A uniform, non‐alternating magnetic field directs lateral motion, and increasing the field strength correlates directly with faster speeds. With an external field of 2 mT, the octopus glides across the surface at 17.4 mm/s.

The aquatic locomoting robotic octopus demonstrates how heterogeneous magnetization can enable complex, multi‐axis motion in soft structures. By isolating actuation to the magnetized arms while keeping the body non‐magnetic, coordinated bending produces effective vertical swimming through water displacement, mimicking the natural behavior of the octopus. The system also exploits passive flotation for lateral surface motion, illustrating that multiple modes of locomotion can be encoded through a combination of geometry, buoyancy, and spatially programmed magnetization. These results highlight the potential of heterogeneously magnetized soft actuators for biomimetic locomotion and multifunctional soft robotic systems, enabling both remote control and adaptable movement in fluidic environments.

#### 2.5.3 Fluidic Serpentine Catheter

Finally, we seek to fabricate a fluidic catheter probe for targeted liquid delivery to the lungs, heart, gastrointestinal tract, or in any surgical environment that requires navigation of a tortuous path. Probes of this nature are currently being explored, but next‐generation designs demand high‐resolution microscale fabrication of flexible hollow structures with programmed 3D magnetization [17, 18]. Our intended design is of a flexible, hollow cylindrical shell in which liquid can pass through. The catheter is designed with discrete magnetic nodes that can be individually targeted with a non‐uniform magnetic field for controlled specific bending. Each node can turn with two degrees of rotational freedom, depending on the external driving field. The magnetization direction is set to the cylinder axis, and each node is magnetized in the same direction under the same conditions during printing. Notably, since the catheter design is hollow, multi‐material, 3D, and requires a specific magnetization direction, it is space‐sensitive to the six magnetic nodes, which in turn require 3D printing with unconstrained, in‐process magnetization. Molding and post‐process magnetization fail, the print direction is impossible to align with the cylinder axis, so printing direction‐based magnetization fails, and the structure is non‐planar, so in‐process magnetization with an external magnet beneath the printing substrate fails.

After fabrication, the hollow probe is manipulated at a distance at the 6 magnetic nodes by cylindrical magnets beneath the probe substrate and manipulated into four simple demonstrative shapes in a single plane (Figure 5h). The entire magnetic catheter is 20 cm long, with 6 nodes, each 5 mm long, separated by non‐magnetic segments, each 10 mm long, and a 400 µm‐wide conical tip printed on the distal magnetic node to allow precise targeted liquid delivery (Figure 5i). The bending behavior is characterized by a single segment of 2 magnetic nodes, with one node fixed and the other allowed to align with an external magnet (Figure 5j). By moving the external magnet in a quarter circle with no rotation in one case, and in a quarter circle while undergoing a rotation of π/2 in the other, two distinct bending patterns emerge. The alignment of the magnetic moment of the free node (${\hat{m}}_{N}$), and of the field direction from the external magnet at the node (${\hat{B}}_{r})$ is calculated as a dot product and recorded as a function of time: $Alignment\;\equiv {\hat{m}}_{N}\cdot {\hat{B}}_{r}$, described more thoroughly in Figure S24. In the first case, where the magnet does not rotate along its quarter‐circle path, the segment falls after bending to ∼31°, as the node cannot mechanically bend to align with the external field. In the second case, however, the external magnet rotates along the path and maintains an alignment between ∼0.76 and ∼1.0 and bends to ∼76°. This simple test demonstrates how complicated the spatial and temporal dynamics of just a single node are in alignment and steering. Aligning 6 nodes, each with 2 degrees of freedom (DOF), in pursuit of a specific bending shape requires more than one field source, which can combine to create a heterogeneous net magnetic field.

To demonstrate the catheter's ability to drive through a tortuous pathway via magnetic stimulation (Figure 5k) and to self‐lubricate and deliver a liquid payload to a difficult‐to‐reach target, we print a mock tissular environment (Figure 5l, full sequence Movie S12). A planar substrate of room‐temperature‐vulcanized (RTV) silicone supports flexible RTV walls, creating a bifurcated structure resembling branching pathways found in the brachial, gastrointestinal, or cardiac systems [18, 51, 52, 53]. A viscous water‐soluble lubricant was mixed with blue dye. It allowed flow via pneumatic pressure through the catheter, which was fed forward manually in place of a typical guidewire and steered at its nodes via an external magnetic field. The friction between the catheter and the rubber walls was reduced via controlled, discrete depositions of the lubricant, which the catheter tip passed through and coated the outer walls as it progressed through the system. When the catheter reached a bifurcation, the first two nodes were manipulated beneath the substrate using magnets, and the correct path to the target was chosen. Upon the magnetic tip reaching the target at the end of its tortuous path, the fluid is changed to water with red dye, the remaining lubricant is deposited, and the red dyed payload is deposited at the target. The stimulating external field for this application in real clinical applications comes from an array of electromagnets free to move in space, called robotic magnetic navigation (RMN) systems. This demonstrates an interesting use case for soft magnetic composites as a relatively non‐invasive microfluidic surgical tool.

## 3 Conclusions

We demonstrate a versatile strategy for programming complex magnetic profiles into 3D‐printed soft materials, enabling precise control over both actuation and sensing behaviors. By integrating an in‐process magnetization system with multi‐material printing, we achieve both discrete and continuous magnetization profiles across a variety of structures. Discretely magnetized solids, such as cubes and meshes, serve as robust platforms for magnetic sensing, providing reproducible Hall sensor responses under compressive, tensile, and bending loads. Continuously magnetized strips and planar structures illustrate how spatially graded magnetization can direct sophisticated bending behaviors, with experimental results closely matching simulations. Extending these principles to biologically inspired actuators, we fabricate a heterogeneously magnetized dragonfly and an aquatic robotic octopus, both of which leverage spatially programmed magnetization to achieve coordinated, multi‐axis motion. The dragonfly demonstrates anisotropic wing bending for alternating actuation, while the octopus achieves vertical swimming through arm displacement and lateral gliding via buoyancy and surface tension. The serpentine catheter represents a complex 3D multimaterial structure with programmable magnetization for high degree of freedom bending and surgical microfluidic applications. These results highlight the ability of continuous, heterogeneous magnetization to encode complex, biomimetic motion in soft structures. Importantly, to isolate the effect of magnetization arising from rotational alignment with a small (∼200 mT) cylindrical magnet, we do not apply a pre‐treatment using a strong (e.g., ∼2 T) saturating impulse field prior to printing. Further investigation is needed to understand the potential synergy between such pre‐treatment and the in‐process magnetization scheme. Overall, this study establishes a robust framework for designing soft, magnetically controlled actuators and sensors with tunable and programmable behaviors. The combination of multi‐material 3D printing and in‐process magnetization opens new possibilities for soft robotics, adaptive structures, and shape‐morphing devices, paving the way for future exploration of multifunctional and biologically inspired magnetic systems.

## 4 Experimental Section

### 4.1 In‐Parallel Process of NdFeB Elastomer

The magnetic elastomer is composed of Dragon Skin parts A and B (Smooth‐On, USA), and magnetic NdFeB microparticles (∼5‐10 µm in diameter, Magnequench, USA). Components were combined manually, then mechanically mixed using a planetary mixer (AR‐100, THINKY, USA) at 1600 rpm for 120 s. The prepared composite was not pretreated with any magnetic field. In some cases, this was explicitly noted before describing the in‐process printing and magnetization method; the composites were then poured into molds to confirm the magneto‐rheology of the composites independent of printing. Square composites used to compare solid‐state versus liquid‐state magnetization were molded and magnetized by proximity to a large external magnet either during or after curing.

All subsequent magnetization was performed in‐process using a freely rotating a neodymium rare earth disc permanent magnet (7.9 mm in diameter × 3.17 mm in thickness, D52‐N52, K & J Magnetics, Inc.,) mounted on a 5 V stepper motor (Adafruit 858) controlled via a ULN2003 stepper driver (ELEGOO^R^) and coordinated with the gantry position through an Arduino Mega R3 2560 programmed in Arduino IDE. NdFeB Elastomer composites were printed on a custom robot gantry (A351, Physik Instrumente L.P.) with full x, y, and z motion control and multiple axes for multi‐material printing. This setup enabled simultaneous deposition of normal and magnetic elastomers while performing in‐process magnetization with the external rotating magnet. Materials were dispensed under pressures ranging from 0.1–300 psi using pneumatic systems (Ultimus V; Nordson EFD; OH, USA).

### 4.2 Magnetic Property Measurements

Magnetic hysteresis was characterized using a Quantum Design VersaLab vibrating sample magnetometer (VSM) with a maximum applied field of 2.5 T. For the first measurement, fully cured samples were tested, while in the second measurement, a 40 wt% NdFeB liquid composite (uncured) was monitored during curing over 1200 s. All measurements were conducted with a field ramp rate of approximately 62.5 mT/s.

### 4.3 Rheology Measurements

Rheological properties were measured using an Anton Paar Modular Compact Rheometer (Anton Paar, Graz, Austria) with a 1 cm diameter parallel plate geometry. Complex viscosity of non‐magnetic elastomeric compounds was recorded either during a 600 s temperature ramp or at fixed temperatures until full curing. Measurements were conducted at a constant shear rate of 10 s^−^^1^ with data collected every 6 s.

### 4.4 Varying Magnetization Conditions via Strip Bending and Magnetic Jumping

To evaluate bending behavior, cured and uncured magnetized square composites were hung vertically while a permanent external magnet approached at a constant velocity of 1 mm/s using the gantry system. Bending angles were quantified using ImageJ and plotted as a function of magnet distance. Magnetic jumping was measured by bringing an external magnet toward droplets on heated or room‐temperature substrates at a fixed speed, recording the distance at which each droplet deformed permanently and contacted the magnet. Droplets of different sizes (2–20 µL) were produced by varying the deposition time using the 3D printing system.

### 4.5 Mechanical Property Measurements

Mechanical testing was carried out using a Texture Analyzer (TA.XT PlusC; Stable Micro Systems). Compressive properties were measured using a 1 cm^2^ stainless steel cylindrical probe, tensile properties were measured with tensile grips, and three‐point bending properties were measured with a standard rig. For all tests, the probe or rig was actuated at a constant speed of 1 mm/s.

### 4.6 Hall Sensor Measurements for Keycard and Actuation

The keycard system consisted of a 3D‐printed keycard reader (Form 2 resin printer, Formlabs) with an RM3100 3‐axis Hall sensor, connected to an ESP32 board programmed with Arduino. A 30 × 30 mm base layer of clear Dragon Skin (Parts A and B) was cast onto a heated bed, and a 4 × 4 array of 5 mm‐diameter circular patterns was printed atop using 40 wt% magnetic ink via a robot gantry (A351, Physik Instrumente L.P.). The circular regions were programmed to be magnetized in arbitrary directions at 60°C using the gantry‐mounted magnet system. Three keycards with distinct magnetization patterns were fabricated, each producing a unique total magnetic field. During operation, the Hall sensor continuously measured *x*, *y*, and *z* magnetic fields. Changes in these fields triggered corresponding ESP32 output pins, which illuminated three LED bulbs according to the detected keycard. Each keycard produced a unique LED pattern based on its magnetization configuration.

For dynamic actuation, the magnetic dragonfly and octopus models were fixed at their centers and actuated under an external solenoid field (1–10 mT peak‐to‐peak) at variable driving frequencies. The dragonfly's wing motion was quantified by overlaying video frames in ImageJ to measure wing arc length over repeated cycles, with front and back wings colored red and blue for clarity. Octopus locomotion was quantified by measuring the time required to swim approximately 10 cm under actuation. For horizontal surface gliding, the solenoid drove the octopus across water at varying magnetic field strengths, and the resulting velocities were recorded.

### 4.7 XRF Measurements

Elemental composition was measured using a PANalytical Epsilon 3XL X‐ray fluorescence spectrometer (PANalytical, Almelo, Netherlands). Samples were analyzed using standardless Omnian screening to determine relative elemental composition.

### 4.8 Micro‐CT Imaging

Microcomputed tomography (micro‐CT) scans were collected using a Bruker SkyScan system (Bruker, Kontich, Belgium). Samples were imaged at a voxel size of 5 µm using an X‐ray source voltage of 120 kV. The reconstructed image stacks were analyzed to visualize the spatial distribution of NdFeB particles within the elastomeric matrix. See Movie S13.

### 4.9 FEA Simulations

FEA simulations were conducted to capture the macroscopic deformation of magneto‐elastomer magnetization under an external magnetic field (Figure s1g and 4b). Simulations were implemented in Python using the FEniCS library. The 3D geometries were modeled in Dassault Systems SolidWorks 2025, and FEA meshes were generated in Gmsh using the Delaunay algorithm with tetrahedral elements and a mesh size factor of 0.1. Mechanical deformation was modeled using Cauchy stress tensor with a linear elastic constitutive law under a small‐strain assumption. Although the structures undergo large overall bending and rotation, the local material strains in the long, thin structures were estimated to remain within approximately 2%–5% during most deformation, with localized regions reaching ∼10%–20% at high‐curvature sections. Under these conditions, the small‐strain approximation provided stable solutions and captured the dominant bending behavior. For comparison, finite deformation using Piola‐Kirchoff stress measures were also evaluated, but produced no meaningful differences in the predicted deformation behavior while substantially increasing computational cost. Therefore, the Cauchy stress formulation was adopted throughout this study.

Magnetic fields were incorporated as body force contributions from local magnetization and external field. The magnetic field was solved independently and coupled one‐way into the mechanical problem, which is a reasonable approximation given the low magnetic permeability contrast and comparatively small deformation relative to the characteristic magnetic field length scale. For simplicity, the magnet was modeled as a clockwise current loop oriented normal to the positive vertical axis, with a radius of 10 mm, and centered over the sample. The loop was positioned at 40 mm, 30 mm, 20 mm, and 10 mm above the material in Figure 1g. For Figure 4b, the loop was translated along the length of the sample in 100‐time steps. The permeability of air is assumed to be ≈ 1.

Simulations were conducted as quasi‐static snapshots, with the current loop translated and rotated in discrete steps; during translation, the loop's normal was rotated clockwise through the length–height plane for one full rotation. Material properties and variables were defined using first‐order continuous Lagrange elements. The FEniCS Newton solver was used with a Krylov solver, LU‐decomposition preconditioning, and the MUMPS factorization solver. Vector fields and magnitudes of the magnetic field and magnetization were visualized using ParaView scripting in Python.

For bending simulations in Figure 2b–i and flower deformation simulations in Figure 4h,i, simulations were implemented in Python using the Torch library. The material was modeled as a rectangular prism (200 mm × 50 mm × 10 mm). Magnetization was defined as constant vectors on the left and right halves of the prism, and the magnetic field was modeled as a point dipole located 100 mm below the prism center, oriented downward. A neural network with 3 input neurons, three fully connected layers of 50 neurons each, and 3 output neurons was trained in three stages: 1000 epochs using AdamW with zero physics loss, 5000 epochs with AdamW including all losses, and 100 epochs with LBFGS including all losses. Deformation was inferred at surface points, which were then translated proportionally to their predicted displacement. The surface mesh was reconstructed via the Delaunay algorithm, and deformation magnitudes were visualized using ParaView scripting in Python.

## Author Contributions

**Phillip Glass**: conceptualization, investigation, writing – original draft, methodology, validation, visualization, writing – review and editing, software, formal analysis. **David Hassouna**: methodology, writing – original draft, software, formal analysis, visualization. **Udena Epitawala Arachchige**: writing – original draft, methodology, visualization, software, formal analysis. **Hyeon Yun Jeong**: writing – review and editing, methodology. **Sung Hyun Park**: conceptualization, funding acquisition, writing – review and editing, methodology. **Daeha Joung**: supervision, funding acquisition, writing – original draft, writing – review and editing, conceptualization, visualization, project administration, methodology.

## Conflicts of Interest

The authors declare no conflict of interest.

## Supporting information




**Supporting File 1**: advs76045‐sup‐0001‐SuppMat.docx.

**Supporting File 2**: advs76045‐sup‐0002‐MovieS1.mp4.

**Supporting File 3**: advs76045‐sup‐0003‐MovieS2.mp4.

**Supporting File 4**: advs76045‐sup‐0004‐MovieS3.mp4.

**Supporting File 5**: advs76045‐sup‐0005‐MovieS4.mp4.

**Supporting File 6**: advs76045‐sup‐0006‐MovieS5.mp4.

**Supporting File 7**: advs76045‐sup‐0007‐MovieS6.mp4.

**Supporting File 8**: advs76045‐sup‐0008‐MovieS7.mp4.

**Supporting File 9**: advs76045‐sup‐0009‐MovieS8.mp4.

**Supporting File 10**: advs76045‐sup‐0010‐MovieS9.mp4.

**Supporting File 11**: advs76045‐sup‐0011‐MovieS10.mp4.

**Supporting File 12**: advs76045‐sup‐0012‐MovieS11.mp4.

**Supporting File 13**: advs76045‐sup‐0013‐MovieS12.mp4.

**Supporting File 14**: advs76045‐sup‐0014‐MovieS13.mp4.

## Data Availability

The data that support the findings of this study are available from the corresponding author upon reasonable request.
