# Supplementary material for: In‐Process Magnetization for 3D Printing of Magnetorheological Elastomer with Heterogeneous Magnetic Profile for Anisotropic Actuation
Source: Adv Sci (Weinh). 2026 Jun 12;13(50):e76045. doi: 10.1002/advs.76045 (PMC13336858; doi:10.1002/advs.76045)
Supplement: Supplementary file 1 [file ADVS-13-e76045-s009.docx]

Supporting Information

In-Process Magnetization for 3D Printing of Magnetorheological Elastomer with Heterogeneous Magnetic Profile for Anisotropic Actuation

Phillip Glass, David Hassouna, Udena Epitawala Arachchige, Hyeon Yun Jeong, Sung Hyun Park, and Daeha Joung*

This Supporting Information includes: comparison of the magnetization methods for programmed ferromagnetic elastomers; real-time infrared measurements of the heated printing bed; time-dependent viscosity of uncured elastomer at fixed curing temperatures; compressive and tensile tests of elastomers with varying NdFeB content after ambient and heat curing; magnetic coercivity versus curing time of a magnetic elastomer; comparison of through-thickness magnetization using below-print and inter-layer magnetization strategies; theoretical framework for modeling the behavior of magnetic microfillers, including analysis of magnetic field–viscosity interactions at fixed temperature, bulk fluid height variations during magnet motion, dimensionless force balance between magnetic and gravitational forces, and time-dependent rotation angle under varying magnet heights; surface magnetic field of elastomers after and during curing; theoretical derivation of the simulated methods and simulation code excerpt; simulated surface fields at varying magnetization heights; computational analysis of bending in a magnetized strip; micro-CT analysis of two composites magnetized at different magnet heights; XRF for samples magnetized at variable height; complex attraction and repulsion behavior of a butterfly-shaped actuator; compressive and tensile cyclability of Hall sensor stray fields in magnetized cube and mesh; schematic of keycard reader setup and LED interface Hall sensor code; resonance modes of an elastomer dragonfly via harmonic finite element analysis; snapshot images of frequency-dependent actuation of eight octopus legs under increasing magnetic field; movie comparing bending angles of cured and uncured magnetized thin composites; movie showing the jumping distance of a magnetic composite droplet; movie of in-process printing and magnetization of a cubic sensor; movie of in-process printing and magnetization of a flexible mesh sensor; movie of compression and tensile stretching of a flexible mesh sensor; movie of a keycard Hall sensor with LED interfaces; movie showing continuous magnetization of thin composites and bending behavior in strips magnetized for 2π and 3π rotations; movie of flower folding upward under an external magnetic field; movie of dragonfly wing actuation at varying driving frequencies; movie of an octopus model exhibiting programmed flapping under an alternating magnetic field; movie of an octopus model exhibiting programmed float on the water surface; and movie of a catheter to drive through a tortuous pathway via magnetic stimulation.

**Table S1.** Tabulation of magnetization methods for programmed ferromagnetic elastomers.

| Method | Magnetizing Source | Curing Method | Process | Magnetization Profile Type | Structure Dimen-sionality | Works |
| --- | --- | --- | --- | --- | --- | --- |
| Uniform Magnetization | Uniform Electromagnet | Ambient | Post-Process | 1-D | 3-D | ^[S1–S4]^ |
| Physically Deformed Uniform Magnetization | Uniform Electromagnet | Ambient | Post-Process | 2-D | 3-D | ^[S5–S12]^ |
| Discrete Element Assembly | Uniform Electromagnet | Ambient | Post-Process | 3-D | 3-D | ^[S13–S15]^ |
| Printing Direction Alignment | Printing Head Electromagnet | Ambient | In-Process | 2-D | 3-D | ^[S16,S17]^ |
| Bottom Mounted External Magnet | Externally Controlled Magnet | Laser, Thermal Printing, etc. | In-Process | 3-D | 2-D | ^[S18,S19]^ |
| Top Mounted External Magnet | Externally Controlled Magnet | Heated Substrate | In-Process | 3-D | 3-D | This Work |

**Figure S1**. Printing Bed Temperature. (a) Infrared temperature measurement of heated bed ramping and cooling to increasing temperatures. The bed was set to ramp to a set temperature to induce elastomer curing (40, 50, 60, and 70° C) and immediately allowed to cool. (b) Infrared measurement of heated bed heating, dwelling, and cooling at increasing temperatures. Here, the bed was set to ramp to a fixed temperature (40, 50, 60, and 70° C) and allowed to maintain the temperature for 200 seconds, and then cooled.

**Figure S2**. Constant Temperature Viscosity Measurement. (a) Elastomer viscosity during curing at increasing fixed temperatures. The viscosity of the uncured non-magnetic elastomer was measured over time at a fixed curing temperature until the composite reached full cure and the viscosity reached a steady state. (b) Rheological measurements repeated for composites with 40 wt% NdFeB confirm that magnetic loading minimally affects temperature-dependent viscosity under a fixed shear rate.

**Figure S3**. NdFeB Weight Percentage Compressive Measurement. Stress-Strain compressive measurement of magnetic elastomer composites of increasing weight percentage NdFeB magnetic filler after (a) ambient curing and (b) heat curing. (c) Compressive Young’s moduli were calculated and tabulated using the previous measurements for increasing weight percentage of NdFeB for ambient and heat-cured composites.

**Figure S4**. NdFeB Weight Percentage Tensile Measurement. Stress-Strain tensile measurement of magnetic elastomer composites of increasing weight percentage NdFeB magnetic filler after (a) ambient curing and (b) heat curing. (c) Tensile Young’s moduli for increasing weight percentage of NdFeB were calculated and tabulated using the previous measurements for ambient and heat-cured composites. (d) Bulk tensile measurement of a standard 'dog-bone' shaped bulk 40 NdFeB elastomer with heat and ambient curing.

**Figure S5**. Time Dependent Coercivity. The width of the hysteresis curve in Figure 1c, the magnetic coercivity, is plotted as a function of curing time. The coercivity of a single composite increasing over time indicates strongly that the propensity to magnetize decreases as the elastomer cures and forms a solid network.

**Figure S6**. Elastomer Surface Field after Cured and Uncured Magnetization. The surface field of ten magnetic solids magnetized after (left) and during (right) curing is given. The two sides of the composite are measured separately, with the top side (red) demonstrating stronger magnetization due to its proximity to the external magnet than the bottom side (blue). Additionally, the liquid elastomer becomes more strongly magnetized than the solid based on the pronounced difference in surface field.

**Figure S7**. Comparison of through-thickness magnetization achieved using (a) magnetization from below and (b) our inter-layer magnetization strategy from above during printing. The axial surface magnetic fields of ten printed magnetic solids are measured at the top (red) and bottom (blue) surfaces after curing. Magnetization from above maintains a more consistent magnet-to-layer distance during fabrication, resulting in stronger magnetic penetration and enhanced top-surface magnetic fields compared to magnetization from below.

**Theoretical Methods**

A theoretical framework for modeling the behavior of the magnetic microfiller under dynamic magnetic field source in a medium of variable viscosity. This theoretical framework helps to predict particle rotation and translation behavior, which inform processing parameters like magnet position and total magnetization time.

*1. General Modeling*

First, we fit the ascending branch of $M\left( B \right)$ to our experimental data:

$$\begin{aligned} M\left( B \right)=M_{S}\tanh\left( \frac{B}{B_{0}} \right)\#\left( 1 \right) \end{aligned}$$

where $M_{S}$, saturation magnetization, and $B_{0}$, a fitting parameter, are both taken from experimental data.

Next, we fit $\eta(t)$ for viscosity during curing at 60° C:

$$\begin{aligned} \chi\left( t \right)={log}_{10}\eta\left( t \right)\#\left( 2 \right) \end{aligned}$$

$$\begin{aligned} \chi\left( t \right)\equiv\left( \eta_{min}+\frac{\eta_{max}-\eta_{min}}{1+e^{-k\left( t-t_{0} \right)}} \right)\#\left( 3 \right) \end{aligned}$$

where $\eta_{min}$, $\eta_{max}$ and fitting parameters $t_{0}$ and k are taken from experimental data.

Next, we model the magnetic field:

$$\begin{aligned} \overset{⃑}{B}\left( x,h \right)=\frac{\mu_{0}m}{4\pi R^{5}}\left( 3\left( \overset{⃑}{m}\cdot\overset{⃑}{r} \right)\overset{⃑}{r}-\overset{⃑}{m}R^{2} \right)\#\left( 4 \right) \end{aligned}$$

and the on-axis field is:

$$\begin{aligned} \overset{⃑}{B}\left( 0,h \right)=\frac{\mu_{0}2m}{4\pi h^{3}}\boldsymbol{\#}\left( 5 \right) \end{aligned}$$

which is fitted to experimental on axis magnetic field data.

*2. Bulk Fluid Model*

We model the magnetically induced upward deformation, jumping, of a viscous magnetic droplet as an overdamped competition between magnetic force and gravity using physically informed values from our general models, and fixed values of volume, viscosity, and hysteresis.

To estimate the net magnetic force, we treat the deformed droplet as a paraboloid of instantaneous height $H$, radius $r_{0}$, and constant volume $V$.

For a paraboloid:

$$\begin{aligned} r(z)^{2}=r_{0}^{2}\left( 1 - \frac{z}{H} \right)\#\left( 6 \right) \end{aligned}$$

and with volume conservation:

$$\begin{aligned} r_{0}^{2}=\frac{2V}{\pi H}\#\left( 7 \right) \end{aligned}$$

So, the differential cross-sectional area at height *z* is therefore:

$$\begin{aligned} dA(z)=\pi r(z)^{2}\text{ }dz\#\left( 8 \right) \end{aligned}$$

Using the local magnetic stress $p_{m}(h_{0}-z)$ where *h_0_* is the initial magnet height, the effective magnetic force is written as:

$$\begin{aligned} F_{m}(H)=\int_{0}^{H} p_{m}(h_{0}-z)\text{ }\pi r(z)^{2}\text{ }dz\#\left( 9 \right) \end{aligned}$$

Numerically, this is evaluated by discretizing the droplet height into $N$ layers of thickness:

$$\begin{aligned} \Delta z_{i}=\frac{H}{N}\#\left( 10 \right) \end{aligned}$$

We chose 50 slices for our simulations.

The total gravitational force is:

$$\begin{aligned} F_{g}=\rho Vg\#\left( 11 \right) \end{aligned}$$

where $\rho$ is the measured droplet density.

Viscous drag taken from Stokes-type hydrodynamic resistance to be

$$\begin{aligned} \zeta=6\pi\eta\left( t \right)r_{0}\#\left( 12 \right) \end{aligned}$$

Then the overdamped velocity is:

$$\begin{aligned} \frac{dH}{dt}=\frac{F_{m}(H)-F_{g}}{\zeta}\#\left( 13 \right) \end{aligned}$$

The jump height can be determined by integrating the overdamped velocity over the chosen cure time by inputting the appropriate magnet trajectory and physical parameters measured from our system.

*3. Particle Migration Model*

The translational motion of magnetic particles within the viscous fluid is modeled as an overdamped balance between magnetic body force and gravity. This formulation captures the migration of particles toward the magnet during curing, which contributes to the spatial redistribution of the magnetic material.

Particles are assumed to be initially well dispersed following synthesis, and migration is studied at the single particle level. The governing equations remain valid in the presence of aggregation; however, aggregation modifies the hydrodynamic drag coefficient by changing the surface geometry, which may introduce a small correction factor to migration distance.

The magnetic force on a particle of volume $V$, volumetric magnetization $M\left( B \right)$, and local field gradient $\nabla B$ is written as

$$\begin{aligned} F_{m}=V(M\left( B \right)+B\frac{dM}{dB})\nabla B\#\left( 14 \right) \end{aligned}$$

The gravitational force for particles of density $\rho_{p}$ in a surrounding fluid of density $\rho_{f}$ is

$$\begin{aligned} F_{g}=\left( \rho_{p}-\rho_{f} \right)Vg\#\left( 15 \right) \end{aligned}$$

We introduce a dimensionless parameter $\Pi(h)$

$$\begin{aligned} \Pi\left( h \right)=\frac{F_{m}}{F_{g}}\#\left( 16 \right) \end{aligned}$$

to investigate which magnet heights encourage either gravitational settling for $\Pi\left( h \right)<1$ or upward particle migration for $\Pi\left( h \right)>1$.

Again, assuming overdamped motion, viscous drag balances the net force:

$$\begin{aligned} F_{m}-F_{g}=F_{drag}\#\left( 17 \right) \end{aligned}$$

Drag is approximated using Stokes resistance for a particle of radius $a$ moving at vertical velocity $v$ as

$$\begin{aligned} F_{drag}=6\pi\eta\left( t \right)av\#\left( 18 \right) \end{aligned}$$

The resulting velocity is

$$\begin{aligned} \frac{dz}{dt}=\frac{V(M\left( B \right)+B\frac{dM}{dB})\nabla B-\left( \rho_{p}-\rho_{f} \right)Vg}{6\pi\eta\left( t \right)a}\#\left( 19 \right) \end{aligned}$$

$$\begin{aligned} \frac{dz}{dt}=\frac{2a^{2}}{9\eta\left( t \right)}((M\left( B \right)+B\frac{dM}{dB})\nabla B-\left( \rho_{p}-\rho_{f} \right)g)\#\left( 20 \right) \end{aligned}$$

*4. Particle Rotation Model*

The rotational alignment of a magnetic particle under an applied magnetic field is modeled as an overdamped balance between magnetic torque and viscous resistance:

$$\begin{aligned} \tau_{M}-\tau_{D}=I\alpha\#\left( 21 \right) \end{aligned}$$

where $\tau_{M}$ and $\tau_{D}$ represent the magnetic and viscous drag torques, respectively.

In the overdamped regime, which applies for the viscosities considered here: (10-10,000 Pa∙s), inertial effects are negligible.

The magnetic torque on a particle of volume *V* with field dependent magnetization *M(B)* is

$$\begin{aligned} \tau_{M}=V M\left( B \right) B\sin\left( \theta\right)\#\left( 22 \right) \end{aligned}$$

The viscous drag torque is written as

$$\begin{aligned} {\tau_{D}=\zeta}_{R}\left( t \right) \left( -\omega\right)\#\left( 23 \right) \end{aligned}$$

where $\zeta_{R}$ is the rotational drag coefficient for a spherical particle of radius *a*

$$\begin{aligned} \zeta_{R}=8\pi\eta\left( t \right)a^{3}\#\left( 24 \right) \end{aligned}$$

We obtain:

$$\begin{aligned} V M\left( B \right) B\sin\left( \theta\right){=\zeta}_{R} \left( -\omega\right)\#(25) \end{aligned}$$

or:

$$\begin{aligned} \omega= \frac{V M\left( B \right) B}{\zeta_{R}}\sin\left( \theta\right)\#\left( 26 \right) \end{aligned}$$

$$\begin{aligned} \frac{d\theta}{\sin\left( \theta\right)}=\frac{-V M\left( B \right)B}{\zeta_{R}}dt\#\left( 27 \right) \end{aligned}$$

$$\begin{aligned} \int\csc\left( \theta\right)d\theta=ln\left| \frac{tan\theta}{2} \right|\#\left( 28 \right) \end{aligned}$$

$$\begin{aligned} ln\left| \frac{tan\theta}{2} \right|=\frac{V M\left( B \right)B}{\zeta_{R}}t+C\#\left( 29 \right) \end{aligned}$$

where $\theta\left( 0 \right)=\frac{\pi}{2}$, $C=0$, and

$$\begin{aligned} \theta\left( t \right)=2{tan}^{-1}\left( e^{-kt} \right)\#\left( 30 \right) \end{aligned}$$

where

$$\begin{aligned} k=\frac{V M\left( B \right)B}{\zeta_{R}}\#\left( 31 \right) \end{aligned}$$

The time required to rotate from 90° to some final angle $\theta_{f}$ is obtained by rearranging the integrated form

$$\begin{aligned} t\left( \theta_{f} \right)=\frac{6\eta}{M\left( B \right)B}\ln\left( \cot\left( \frac{\theta_{f}}{2} \right) \right)\#\left( 32 \right) \end{aligned}$$

**Figure S8**. Physically informed models of (a) axial B field, (b) viscosity at fixed temperature, and (c) the ascending branch of magnetic hysteresis. Experiment and fit are given in black and red respectively.

**Figure S9**. Theoretical plot of bulk fluid height as a function of time during descending magnet trajectory for (a) variable volume and (b) viscosity.

**Figure S10**. (a) Theoretical plot of the dimensionless force competition between magnetic force *F_m_*, and gravitational force *F_g_ .* (b) Migration distance after 60 seconds at fixed viscosity.

**Figure S11**. Theoretical plot of rotation angle as a function of time at variable magnet height at fixed viscosity, and variable viscosity at fixed magnet height.

**Simulation Methods**

Theoretical derivations of the materials' properties have been used for simulation throughout the manuscript.

*1. Maxwell’s Equations*

The magnetic behavior of the material is governed by Maxwell’s macroscopic equations, considered over a domain $\Omega\subset R^{3}$. These equations form the foundation for constructing a variational problem in the Hilbert space $V\subset H^{1\left( \Omega^{3} \right)}$:

$\begin{aligned} \nabla\cdot D=\rho_{f}\#\left( 33 \right) \\ \nabla\cdot B=0\#\left( 34 \right) \\ \nabla\times E=-\frac{\partial B}{\partial t}\#\left( 35 \right) \\ \nabla\times H=J_{f}+\frac{\partial D}{\partial t}\#\left( 36 \right) \end{aligned}$ where *D* is the electric displacement field, *ρ_f_* is the free charge density, *B* is the magnetic flux density, *E* is the electric field, *H* is the magnetizing field, and *J_f_* is the free current density.

Defining the vector potential *A* as:

$$\begin{aligned} B=\nabla\times A\#\left( 37 \right) \\ \nabla\cdot A=0\#\left( 38 \right) \\ H=\frac{1}{\mu_{0}}B+M\#\left( 39 \right) \end{aligned}$$

, where 𝜇_0_ is the magnetic permeability of free space and *M* is the magnetization, and assuming pseudo–steady state conditions, the governing equation simplifies to:

$$\begin{aligned} \frac{1}{\mu_{0}}\nabla\times\nabla\times A=-\nabla\times M+J_{f}\#\left( 40 \right) \end{aligned}$$

Using integration by parts and the identity

$$\begin{aligned} \nabla^{2}A=\nabla\left( \nabla\cdot A \right)-\nabla\times\nabla\times A\#\left( 41 \right) \end{aligned}$$

the equation becomes:

$$\begin{aligned} \frac{1}{\mu_{0}}\nabla^{2}A=\nabla\times M-J_{f} \#\left( 42 \right) \end{aligned}$$

This represents the **strong form** of the magnetic constitutive equation.

$$\begin{aligned} \left\langle au,\nu\right\rangle=\left\langle L,\nu\right\rangle\#\left( 43 \right) \end{aligned}$$

By integrating against a test function *v ∈ V_i_*, the **weak form** is obtained:

$$\begin{aligned} \frac{1}{\mu_{0}}\int_{\Omega} \nu\cdot\nabla^{2}A d\Omega=\int_{\Omega} \nu\cdot\nabla\times Md\Omega-\int_{\Omega} \nu\cdot J_{f}d\Omega\#\left( 44 \right) \end{aligned}$$

The second order derivative is reduced by the product rule for divergence, the curl of a curl identity, and the divergence theorem, giving a variant of Green's first identity

$$\begin{aligned} \nabla\cdot\left( FG \right)=\left( \nabla\cdot F \right)G+\left( F\cdot\nabla\right)G\#\left( 45 \right) \\ \nabla\times\left( \nabla\times F \right)=\nabla\left( \nabla\cdot F \right)-\nabla^{2} F\#\left( 46 \right) \\ \int_{\Omega} \nabla\cdot Fd\Omega=\oint_{\partial\Omega} F\cdot dS\#\left( 47 \right) \\ \int_{\Omega} G\cdot\nabla^{2}Fd\Omega+\int_{\Omega} \nabla F:\nabla Gd\Omega=\oint_{\partial\Omega} G\cdot\left( \nabla F\cdot dS \right)\#\left( 48 \right) \end{aligned}$$

Where *F* and *G* are arbitrary vector fields, and $\Omega$ is some region $\Omega\subset V$, $\partial\Omega$ is the boundary of the region $\Omega$, and $dS$ is the outward-pointing oriented surface element.

Applying the divergence theorem and the Coulomb gauge condition *∇⋅A=0* yields:

$$\begin{aligned} \int_{\Omega} \nu\cdot\nabla^{2}A d\Omega+\int_{\Omega} \nabla\nu:\nabla A d\Omega=\oint_{\partial\Omega} \nu\cdot\left( \nabla A\cdot dS \right)\#\left( 49 \right) \\ \int_{\Omega} \nu\cdot\nabla^{2}A d\Omega=-\int_{\Omega} \nabla\nu:\nabla A d\Omega\#\left( 50 \right) \\ \frac{1}{\mu_{0}}\int_{\Omega} \nabla\nu:\nabla A d\Omega=\int_{\Omega} \left( \nabla\times M \right)\cdot\nu d\Omega-\int_{\Omega} J_{f}\cdot\nu d\Omega\#\left( 51 \right) \end{aligned}$$

The bilinear (*a*) and linear (*L*) forms are thus defined as:

$$\begin{aligned} a=\int_{\Omega} \frac{1}{\mu_{0}}\nabla u:\nabla v d\Omega\#\left( 52 \right) \end{aligned}$$

$$\begin{aligned} L=\int_{\Omega} \left( \nabla\times M \right)\cdot\nu d\Omega-\int_{\Omega} J_{f}\cdot\nu d\Omega\#\left( 53 \right) \end{aligned}$$

The resulting algebraic form
$\begin{aligned} a\mathbb{x=}L\#\left( 54 \right) \end{aligned}$is solved using the generalized minimal residual (GMRES) Krylov subspace method.

*2. Elasticity: Cauchy Stress and Strain*

Mechanical deformation follows the static Cauchy momentum equation, Hooke’s law for isotropic linear elasticity, and the definition of the symmetric strain tensor:

$$\begin{aligned} -\nabla\cdot\sigma=f\#\left( 55 \right) \\ \sigma=\lambda tr\left( \epsilon\right)I+2\mu\epsilon\#\left( 56 \right) \\ \epsilon=\frac{1}{2}\left( \nabla u+\left( \nabla u \right)^{T} \right)\#\left( 57 \right) \end{aligned}$$

where σ is the Cauchy stress tensor, *f* is the body force per unit volume, *λ* and *μ* are the Lamé parameters, *tr* is the trace operation, $\epsilon$ is the symmetric strain tensor, $I$ is the identity tensor, and $u$ is the deformation field.

Combining these relations gives:

$$\begin{aligned} \sigma=\lambda\left( \nabla\cdot u \right)I+\mu\left( \nabla u+\left( \nabla u \right)^{T} \right)\#\left( 58 \right) \end{aligned}$$

The inner product with a test function is

$$\begin{aligned} -\int_{\Omega} \nu\cdot\left( \nabla\cdot\sigma\right)d\Omega=\int_{\Omega} \nu\cdot fd\Omega\#\left( 59 \right) \end{aligned}$$

, which has second-order derivatives with respect to $u$. Assuming $\sigma$ is 0 on the external boundary, integration by parts is used to linearize it

$$\begin{aligned} \int_{\Omega} \nu\cdot\left( \nabla\cdot\sigma\right)d\Omega=\int_{\Omega} \sigma:\nabla\nu d\Omega-\oint_{\partial\Omega} \nu\cdot\left( \sigma\cdot n \right)dS\#(60) \\ \int_{\Omega} \nabla\nu:\sigma d\Omega=\int_{\Omega} \nu\cdot fd\Omega+\oint_{\partial\Omega} \nu\cdot\left( \sigma\cdot n \right)dS\#(61) \end{aligned}$$

The Cauchy stress tensor is defined by

$$\begin{aligned} T^{\left( n \right)}=n\cdot\sigma\#\left( 62 \right) \end{aligned}$$

where $n$ is the surface normal, and $T^{\left( n \right)}$ is the traction vector through the surface element perpendicular to $n$. Leading to

$$\begin{aligned} \begin{aligned} \int_{\Omega} \nabla\nu:\sigma d\Omega=\int_{\Omega} \nu\cdot fd\Omega+\oint_{\partial\Omega} \nu\cdot TdS\#\left( 63 \right) \end{aligned} \end{aligned}$$

The resulting weak form eq (11) is

$$\begin{aligned} a=\int_{\Omega} \epsilon\left( \nu\right):\sigma\left( u \right)d\Omega\#\left( 64 \right) \\ \sigma=\lambda\left( \nabla\cdot u \right)I+\mu\left( \nabla u+\left( \nabla u \right)^{T} \right)\#\left( 65 \right) \\ \epsilon=\frac{1}{2}\left( \nabla\nu+\left( \nabla\nu\right)^{T} \right)\#\left( 66 \right) \\ L=\int_{\Omega} \nu\cdot fd\Omega+\oint_{\partial\Omega} \nu\cdot TdS\#\left( 67 \right) \end{aligned}$$

The equation for a magnetic dipole point source is

$$\begin{aligned} B=\frac{\mu_{0}}{4\pi}\left( \frac{3r\left( m\cdot r \right)}{r^{5}}-\frac{m}{r^{3}} \right)\#\left( 68 \right) \end{aligned}$$

, where $m$ is the magnetic dipole moment, and $r$ is the distance from the current point to the source point. And the magnetic body force is given by

$$\begin{aligned} f=\nabla\left( m\cdot B \right)\#\left( 69 \right) \end{aligned}$$

*3. Magnetic Hysteresis Model*

To capture nonlinear magnetization behavior, a modified Preisach hysteresis model was implemented at each finite element. Given that the applied external magnetic field is small relative to the material’s remanent magnetization and the material is initially demagnetized, the local hysteresis curve is linearized near the origin with slope *C_m_*.

Each element is assigned a modified hysteron governed by:

$$\begin{aligned} M\left( B_{ext} \right)=\left\{ \begin{aligned} M_{t-1}, &C_{m}B_{ext}<H_{c} \\ C_{m}B, &H_{c}\leq C_{m}B_{ext} andM_{t-1}\leq C_{m}B_{ext} \end{aligned} \right.\#\left( 70 \right) \end{aligned}$$

where *M_t-1_* is the magnetization from the previous timestep, *H_c_* is the coercive field, and *B_ex_*_t_ is the local external magnetic field.

**Simulation 1. Effect of Magnetization Height (for Figure 1g)**

The simulation was implemented in Python using the FEniCS library. The sample was modeled as a cylindrical elastomer (length = 40 mm, radius = 5 mm) surrounded by an air domain represented as a rectangular prism (50 mm × 30 mm × 40 mm). The geometry was created in Dassault Systèmes SolidWorks 2025, exported as a STEP file, and meshed in Gmsh using the Delaunay algorithm with tetrahedral elements (mesh size = 0.1). The resulting finite-element mesh contained 120,961 vertices, 822,809 edges, 1,378,615 faces, and 676,766 cells.

A clockwise current loop, oriented normal to the positive vertical axis and centered above the sample, was used to generate the magnetic field. The loop had a radius of 10 mm and was positioned at heights of 40 mm, 30 mm, 20 mm, and 10 mm above the sample. All equations were expressed in SI units and nondimensionalized prior to computation.

The magnetic vector potential field was computed using equations (**52**) and (**53**), with all field variables and material properties defined using first-order continuous Lagrange elements. The gauge condition was enforced by applying *A=0* on the outer surfaces of the air domain, and continuity of A was imposed across all internal boundaries. The nonlinear FEniCS Newton solver employed a Krylov–LU scheme with MUMPS factorization, a relative tolerance of 1×10^−9^, and an absolute tolerance of 1×10^−10^. Convergence was typically achieved within three iterations. The magnetic field and magnetization were subsequently obtained from equations (**37**) and (**68**), respectively. Additionally, the magnetization was modified according to equation (**70**). Vector field visualizations and magnitude plots were generated in ParaView using Python scripting, with current and permeability values scaled for visual clarity.

**Simulation 2. Deformation from Dual-Region Magnetization (for Figure 2b-i)**

A second simulation investigated magneto-mechanical deformation in a soft sample with distinct magnetization orientations in two regions. The sample was modeled as a rectangular prism (200 mm × 50 mm × 10 mm) and meshed directly in FEniCS using a structured grid (100 × 6 × 6 cells), forming 4,949 vertices, 29,020 edges, 45,672 faces, and 21,600 cells. The left and right halves of the sample were assigned constant magnetization vectors pointing right, left, up, or down, resulting in 16 orientation combinations. The external magnetic field was modeled as a point dipole located 100 mm below the sample center, oriented downward. Deformation was computed using equations (**64**–**67**) with first-order Lagrange elements.

Boundary conditions enforced zero traction on outer surfaces and fixed displacement at the sample’s mid-length. The linear solver used LU preconditioning and MUMPS factorization with the same tolerance criteria as prior simulations. Mesh nodes were displaced according to computed deformation fields, and ParaView visualization displayed deformation magnitudes on the deformed geometry. Material parameters (Lamé constants, magnetization, and field strength) were scaled for clarity in the resulting figures.

**Simulation 3. Spatially Varying Magnetization Orientation (for Figure 4b)**

This simulation, also performed in FEniCS, modeled a cylindrical sample (length = 1000 mm, radius = 10 mm) within an air domain of 1040 mm × 60 mm × 100 mm. The geometry was designed in SolidWorks 2025, meshed in Gmsh (mesh size = 0.05), and comprised 484,319 vertices, 3,287,800 edges, 5,503,192 faces, and 2,699,710 cells. A clockwise current loop (radius = 10 mm) initially normal to the vertical axis was centered over the left side of the sample and translated along its length in 100 quasi-static steps. Simultaneously, the loop’s normal vector rotated clockwise through the length–height plane, completing one full revolution during translation.

All variables were expressed in SI units and normalized before entry into the governing equations. The magnetic vector potential was solved using equations (**52**) and (**53**) with first-order Lagrange elements. The boundary condition *A=0* was applied to all exterior facets, ensuring continuity across internal boundaries. A linear FEniCS solver with LU preconditioning and MUMPS factorization was used, with relative and absolute tolerances of 1×10^−9^ and 1×10^−10^, respectively. The resulting magnetic field and magnetization were computed from equations (**37**) and (**68**). Additionally, the magnetization was modified according to Equation (**70**). Visualization of field distributions and vector magnitudes was performed in ParaView, with current and permeability values scaled to enhance contrast.

**Simulation 4. Deformation with Spatially Varying Magnetization (for Figure S6)**

The final simulation examined deformation in a sample (200 mm × 50 mm × 10 mm) with spatially varying magnetization. The mesh, identical to the one in Figures 2b-i Simulations, comprised 4,949 vertices and 21,600 cells. Magnetization was modeled as a sinusoidal function in both the length and height directions, representing the field pattern generated by a rotating magnet translating along the sample.

Simulations were conducted for magnetization periods corresponding to 0.25, 0.5, 1, 1.5, 2, 2.5, and 3 full rotations. The external magnetic field was oriented upward and varied linearly with height. Deformation was computed using equations (**64**–**67**) with zero traction boundary conditions and fixed displacements at both ends of the sample. The FEniCS linear solver used LU–MUMPS factorization with relative and absolute tolerances of 1×10^−9^ and 1×10^−10^, respectively. The deformed mesh and displacement magnitudes were visualized in ParaView, with material parameters scaled to highlight deformation gradients.

**Simulation 5. Deformation of flower with programmed magnetization orientation (for Figure 4h,i)**

The deformation simulation of the flower structure shown in Figure 4 was implemented in Python using the Torch library. The material was modeled as a rectangular prism with dimensions of 200 mm × 200 mm × 1 mm. Magnetization was defined as a constant vector oriented differently across the inner and outer petals. At the same time, the external magnetic field was represented by a point magnetic dipole located 100 mm above the sample's center and oriented downward. The physics loss was formulated following equations (**58**) and (**69**).

The neural network architecture included three input neurons, three fully connected layers with 50 neurons each, and three output neurons. Training was conducted in three stages: (I) 1000 epochs using the AdamW optimizer with zero physics loss, (II) 5000 epochs using AdamW with all losses included, and (III) a final 100 epochs using the LBFGS optimizer with all losses active. After training, the model was used to infer displacements on the prism’s surface points to simulate deformation. The displaced surface points were translated proportionally to their predicted deformations, reconstructed using the Delaunay triangulation algorithm, and visualized in ParaView through Python scripting, which generated deformation magnitude maps on the deformed mesh.

```
# IMPORT MODEL
mff.step_to_msh(filename, step_path, mesh_path, size=mesh_size, log=False)
mff.msh_to_xdmf(filename, mesh_path)
mesh, ct, pt = mff.import_xdmf(filename, mesh_path, comm, rank)

# SET UP OUTPUT FILE
file = dolfinx.io.VTKFile(comm, output_path + filename + "_output.pvd", 'w')

# DEFINE CONSTANT FIELD VARIABLES
V = dolfinx.fem.functionspace(mesh, ("Lagrange", 1, (3,)))
Mtemp = dolfinx.fem.Function(V)
M = dolfinx.fem.Function(V)
M.interpolate(Mtemp, ct.find(2))

while t <= tf:

    # DEFINE FIELD VARIABLES
    mu = mff.const_to_f(V, (mu_, mu_, mu_))
    Cm = mff.const_to_f(V, (Cm_, Cm_, Cm_), cells=ct.find(noodle))
    J = dolfinx.fem.Function(V) #virtual current in permanent magnet
    J = mff.python_func_to_f(J_expr, V, ct.find(background))
    magnet_position_and_direction())

    # BOUNDARY CONDITIONS
    ext_facets = mff.getExternalFacets(mesh, ct, 3)
    external_bcs = mff.set_bcs_const(V, mesh, ext_facets, (0.0, 0.0, 0.0))
    bcs.append(external_bcs)

    # ASSEMBLE FORMS
    A = dolfinx.fem.Function(V)
    v = ufl.TestFunction(V)
    c = -1.0 * (ufl.sqrt(ufl.dot(Cm, Cm)) + ufl.sqrt(ufl.dot(mu, mu)))
    a = c * ufl.inner(ufl.curl(A), ufl.curl(v)) * ufl.dx
    L = ufl.dot(J, v) * ufl.dx
    F = a - L
    problem = dolfinx.fem.petsc.NonlinearProblem(F, A, bcs=bcs)
    solver = dolfinx.nls.petsc.NewtonSolver(mesh.comm, problem)
    num_iterations, converged = solver.solve(A)

    # CALCULATIONS
    B = mff.ufl_to_f(ufl.curl(A), V)
    H_expr_ufl = (1/ufl.sqrt(ufl.dot(mu, mu)))*B-ufl.sqrt(ufl.dot(Cm, Cm))*B
    H = mff.ufl_to_f(H_expr_ufl, V)

    # SAVE DATA
    mff.save_to_vtk(file, t, [("Cm", Cm), ("B", B), ("M", M)])
    t += delta_t

file.close()
```

**Figure S12a**. Magnetization Simulation Code Excerpt. Magnetization Simulation Code Excerpt. Condensed code that imports an stl file, and defines field variables and boundary conditions. Then the FEA weak form for the magnetic vector potential is assembled into a matrix and solved. Finally, the magnetic B and H fields are calculated and saved to a vtk file. We used these codes for Figures 1g and 4b.

bc_types = ["middle", "both_ends"]

```
num_magnet_rotations = [0.25, 0.5, 1.0, 1.5, 2.0, 2.5, 3]
domain = dolfinx.mesh.create_box(comm, [np.array([0, 0, 0]), np.array([Length, Height, Width])], [100, 6, 6], cell_type=dolfinx.mesh.CellType.tetrahedron)
V = dolfinx.fem.functionspace(domain, ("Lagrange", 1, (3, )))
Q = dolfinx.fem.functionspace(domain, ("DG", 0, (3,)))

# DEFINE FIELDS
if bc_type == "middle":
    def clamped_boundary(x):
        return np.isclose(x[0], 100)
if bc_type == "both_ends":
    def clamped_boundary(x):
        a = np.full_like(x[0], fill_value=False, dtype=bool)
        tol = 1e-3
        for i in range(x[0].size):
            if abs(x[0][i]) < tol or abs(200 - x[0][i]) < tol:
                a[i] = True
        return a

boundary_facets = dolfinx.mesh.locate_entities(domain, fdim, clamped_boundary)
u_D = np.array([0, 0, 0], dtype=dolfinx.default_scalar_type)
bc = dolfinx.fem.dirichletbc(u_D, dolfinx.fem.locate_dofs_topological(V, fdim, boundary_facets), V)
T = dolfinx.fem.Constant(domain, dolfinx.default_scalar_type((0, 0, 0)))
B = mff.python_func_to_f(find_B_at_point, V)
M = mff.python_func_to_f(find_M_at_point, V)

# ASSEMBLE FORMS
def epsilon(u): return ufl.sym(ufl.grad(u))
def sigma(u):return ufl.nabla_div(u)*ufl.Identity(len(u))+2*mu[i]*epsilon(u)

ds = ufl.Measure("ds", domain=domain)
u = ufl.TrialFunction(V)
v = ufl.TestFunction(V)
a = ufl.inner(sigma(u), epsilon(v)) * ufl.dx
f = ufl.grad(ufl.dot(M, B))
L = ufl.dot(f, v) * ufl.dx + ufl.dot(T, v) * ds

problem = dolfinx.fem.petsc.LinearProblem(a, L, bcs=[bc], petsc_options={"ksp_type": "preonly", "pc_type": "lu"})#
uh = problem.solve()
fmag = mff.ufl_to_f(f, V)

# SAVE DATA
mff.save_to_vtk_with_mesh(file,t,domain,[("B",B),("M",M),("F",fmag),("uh",uh)])
file.close()
paraview_plot_deformation(filename+"_"+bc_type+"_"+numstr, output_path)
```

**Figure S12b**. Deformation Simulation Code Excerpt. Deformation Simulation Code Excerpt. Condensed code that imports an stl file, and defines field variables and boundary conditions. Then the FEA weak form for the deformation is assembled into a matrix and solved. Finally, the applied magnetic force field is calculated and saved to a vtk file. We used these codes for Figure 2b-I.

```
# ========== LOAD AND PREPARE DATA ==========
df = pd.read_csv(data_path+".csv")
pts = df.to_numpy()
X_data, y_data, pvxdata, pvydata = process_raw_data(pts, plot=False)

# PINN
def physics_loss(model, pts)
def boundary_loss(model, X_bc, u_bc)
model_config = {}
net = pinn.Net(**model_config).to(device)
net.load_checkpoint(checkpoint_path)

# TRAIN
trainer_config1 = {
    "data_loss": nn.MSELoss(),
    "physics_loss": physics_loss,
    "boundary_loss": boundary_loss}
adamtrainer1 = pinntrainer.Trainer(net, **trainer_config1)
losses = adamtrainer1.fit(1000, X_data=X_data, y_data=y_data)

trainer_config2 = {
    "data_loss": nn.MSELoss(),
    "physics_loss": physics_loss,
    "boundary_loss": boundary_loss}
adamtrainer2 = pinntrainer.Trainer(net, **trainer_config2)
losses = adamtrainer2.fit(5000, X_data=X_data, y_data=y_data)

trainer_config3 = {
    "data_loss": nn.MSELoss(),
    "physics_loss": physics_loss,
    "boundary_loss": boundary_loss}
lbfgstrainer = pinntrainer.Trainer(net, **trainer_config3)
losses = lbfgstrainer.fit(100, X_data=X_data, y_data=y_data)
net.save_checkpoint(checkpoint_path)

# PREDICT DISPLACEMENT FIELD
points = generate_equidistant_surface_points(length=Length, width=Width, height=abs(Height), num_points=1000)
u_pred = net.predict(points)


# PLOTTING
```

**Figure S12c**. PINN Simulation Code Excerpt. A csv of a few experimentally determined datapoints is loaded. Then the physics loss, boundary loss, and model configuration are defined. Next, a three-phase training scheme is applied using the AdamW and LBFGS trainers from PyTorch. Finally, the network is evaluated to predict the deformation of a set of surface points, and the points are plotted. We used these codes for Figure 4h.

**Figure S13**. FEA Simulated Magnetization Height Surface Field. The surface field of magnetized composites magnetized from an external magnet at a variable height. The behavior and shape of the simulated data closely match those of the experimental results in Figure 1h.

**Figure S14**. (a). X-Ray Fluoresence for samples magnetized at variable height, and bisected to investigate migration via compositional change. (b) Measured weight percentage per sample and (c) as a function of magnet height.

**Figure S15**. Micro-CT analysis of two composites magnetized at different magnet heights. For the sample magnetized at a height of ~10 mm, the composite demonstrates moderate particle aggregation but no significant lateral or vertical migration, as evidenced by the relatively uniform particle distribution and histogram of average pixel intensity as a function of height (a–c). In contrast, the sample magnetized at ~0 mm exhibits particle aggregation together with noticeable vertical redistribution under the strong magnetic field gradient, while still showing minimal lateral migration (d–f). The increased particle density near the upper surface is reflected in the corresponding pixel intensity profile, indicating upward particle migration during curing.

**Figure S16**. Complex Attraction and Repulsion Behavior. For a butterfly (a) magnetized uniformly and fixed about its center, two magnetic regions are printed on each side at equal volume. Under an upward external field (b) both sides of the structure bend upwards along the direction of the external field. Under an opposite, downward field, however, the right side of the structure experiences a repulsion, and the left side twists and demonstrates a competitive attraction from its underside.

**Figure S17**. Compressive Cyclability of a 3D Cube Sensor. Hall sensor stray-field and mechanical measurements of magnetized mesh under 2,000 cycles of tension. The stray field measurement temporally aligns well with the compression measurement, indicating no lag or hysteresis. Both sets of measurements are completely stable for 2,000 cycles, demonstrating no significant mechanical or magnetic changes throughout the measurement.

**Figure S18**. Tensile Cyclability of a Mesh Sensor. Hall sensor stray-field and mechanical measurements of magnetized mesh under 2,000 cycles of tension. The stray field measurement synchronizes well temporally with the compression measurement, indicating no lag or hysteresis. Both sets of measurements are completely stable for 2,000 cycles, demonstrating no significant mechanical or magnetic change over the entirety of the measurement.

**Figure S19**. Keycard Schematic. A keycard reader (a) is 3D printed, which has a (b) 3-axis Hall sensor reading. When a (c) 3D printed keycard with 16 magnetized pixels is inserted into the slot, the Hall sensor is (d) programmed to read the stray field, and light a combination of LEDs depending on which keycard is inserted. (e) Each keycard has a unique signal and LED combination.

```
#include <SPI.h>
// RM3100 Register Addresses
#define CMM  0x01
#define CCX  0x04
#define CCY  0x06
#define CCZ  0x08
#define TMRC 0x0B
#define POLL 0x00
#define MXYZ 0x24

#define CS_PIN 5
#define LED_PIN_1 15
#define LED_PIN_2 2
#define LED_PIN_3 4

float x_offset = 0;
float y_offset = 0;
float z_offset = 0;

void writeRegister(uint8_t reg, uint8_t val) {
  digitalWrite(CS_PIN, LOW);
  SPI.transfer(reg | 0x80);
  SPI.transfer(val);
  digitalWrite(CS_PIN, HIGH);
}

void writeRegister24(uint8_t reg, uint32_t val) {
  digitalWrite(CS_PIN, LOW);
  SPI.transfer(reg | 0x80);
  SPI.transfer((val >> 16) & 0xFF);
  SPI.transfer((val >> 8) & 0xFF);
  SPI.transfer(val & 0xFF);
  digitalWrite(CS_PIN, HIGH);
}

void startMeasurement() {
  digitalWrite(CS_PIN, LOW);
  SPI.transfer(POLL);
  SPI.transfer(0x70);
  digitalWrite(CS_PIN, HIGH);
}

int32_t readAxis(uint8_t reg) {
  digitalWrite(CS_PIN, LOW);
  SPI.transfer(reg);
  uint8_t b1 = SPI.transfer(0x00);
  uint8_t b2 = SPI.transfer(0x00);
  uint8_t b3 = SPI.transfer(0x00);
  digitalWrite(CS_PIN, HIGH);

  int32_t val = ((int32_t)b1 << 16) | ((int32_t)b2 << 8) | b3;
  if (val & 0x800000) val |= 0xFF000000;
  return val;
}

void setup() {
  Serial.begin(115200);
  SPI.begin();
  pinMode(CS_PIN, OUTPUT);
  digitalWrite(CS_PIN, HIGH);

  pinMode(LED_PIN_1, OUTPUT);
  pinMode(LED_PIN_2, OUTPUT);
  pinMode(LED_PIN_3, OUTPUT);
  digitalWrite(LED_PIN_1, LOW);
  digitalWrite(LED_PIN_2, LOW);
  digitalWrite(LED_PIN_3, LOW);

  delay(100);

  writeRegister24(CCX, 200);
  writeRegister24(CCY, 200);
  writeRegister24(CCZ, 200);

  writeRegister(CMM, 0x00);
  writeRegister(TMRC, 0x92);

  // Take initial reading for tare
  delay(100);
  startMeasurement();
  delay(10);
  x_offset = readAxis(MXYZ) * 0.075;
  y_offset = readAxis(MXYZ + 3) * 0.075;
  z_offset = readAxis(MXYZ + 6) * 0.075;

  Serial.println("Sensor tared:");
  Serial.print("X offset: "); Serial.println(x_offset);
  Serial.print("Y offset: "); Serial.println(y_offset);
  Serial.print("Z offset: "); Serial.println(z_offset);
}

void loop() {
  startMeasurement();
  delay(10);

  int32_t x = readAxis(MXYZ);
  int32_t y = readAxis(MXYZ + 3);
  int32_t z = readAxis(MXYZ + 6);

  float x_uT = x * 0.075 - x_offset;
  float y_uT = y * 0.075 - y_offset;
  float z_uT = z * 0.075 - z_offset;

  Serial.print(x_uT); Serial.print(",");
  Serial.print(y_uT); Serial.print(",");
  Serial.println(z_uT);

  // LED 1: Range Check
  if (x_uT >= -55 && x_uT <= -45 &&
      y_uT >= -5 && y_uT <= 2 &&
      z_uT >= -9 && z_uT <= 3) {
    digitalWrite(LED_PIN_1, HIGH);
    digitalWrite(LED_PIN_2, LOW);
    digitalWrite(LED_PIN_3, LOW);
  }

  // LED 2: Range Check
  else if (x_uT >= 15 && x_uT <= 22 &&
      y_uT >= 5 && y_uT <= 15 &&
      z_uT >= -15 && z_uT <= -5) {
    digitalWrite(LED_PIN_2, HIGH);
    digitalWrite(LED_PIN_1, LOW);
    digitalWrite(LED_PIN_3, LOW);
  }

  // LED 3: Range Check
  else if (x_uT >= -20 && x_uT <= -14 &&
      y_uT >= -3 && y_uT <= 2 &&
      z_uT >= -2 && z_uT <= 4) {
    digitalWrite(LED_PIN_3, HIGH);
    digitalWrite(LED_PIN_1, LOW);
    digitalWrite(LED_PIN_2, LOW);
  }

  else {
  digitalWrite(LED_PIN_1, LOW);
  digitalWrite(LED_PIN_2, LOW);
  digitalWrite(LED_PIN_3, LOW);
}

  delay(100);
}
```

**Figure S20**. Hall Sensor LED Code.

**Figure S21**. Computational Analysis of the Bending of a Magnetized Strip fixed on both ends, in which a magnet is allowed to rotate *N* times over the length of the strip in the length-height plane. One rotation, for example, imparts downwards magnetization at *L*=0 and *L*=*L*, and upwards magnetization direction at *L*=*L*/2. The specific way the composites are magnetized produces significantly different bending behaviors, as shown in the simulation.

**Figure S22**. Resonance modes of an elastomer dragonfly, modeled after the 3D printed structure. Using harmonic finite element analysis, the resonant vibration modes of the actuation of the front and back wings as they undergo opposite responses (attractive and repulsive) to two external fields going right to left (a) and left to right (b). The stress is plotted as a color map from 0 to 1 MPa.

**Figure S23**. Frequency-dependent actuation of eight octopus’ legs under increasing driving magnetic field frequency. Movies are taken for each driving frequency (full sequence is shown in Movie S10), and the frames are overlaid to demonstrate the entire path of the magnetized tentacles.

**Alignment dot product calculation**

Below is the derivation of a dimensionless value which describes the alignment of the magnetic moment of the free magnetic node ($\hat{m}_{N}$), the magnetic moment of the external magnets ($\hat{m}_{M}$), and the magnetic field at the node ($\hat{B}_{r}$) from the external rotating magnet:

The dot product of these two unit vectors describes how well aligned:

$$Alignment\equiv\hat{m}_{N}\cdot\hat{B}(\hat{r})$$

, where

$$B\left( r \right)=\frac{\mu_{0}}{4\pi r^{3}}(3\left( m_{M}\cdot\hat{r} \right)\hat{r}-m_{M})$$

And therefore made into a unit direction vector:

$$\hat{B}\left( \hat{r} \right)=\frac{(3\left( m_{M}\cdot\hat{r} \right)\hat{r}-m_{M})}{\left\| (3\left( m_{M}\cdot\hat{r} \right)\hat{r}-m_{M}) \right\|}$$

Since $\hat{m}_{N}$ and $\hat{m}_{M}$ are measurable, the alignment coefficient, external magnet angle, and magnetic node angle are plotted as a function of time.

**Figure S24**. Photo image of an alignment of the magnetic moment of the free magnetic node ($\hat{m}_{N}$), the magnetic moment of the external magnets ($\hat{m}_{M}$) and the magnetic field at the node from the external rotating magnet ($\hat{B}_{r}$).

**References**

[S1]	Z. Peng, H. Zhang, M. Wang, S. Zhang, Y. Jiang, Y. Li, X. Zhu, G. Zhang, G. Niu, J. Zhang, J. He, H. Lan, *Adv. Mater. Technol.* **2024**, *9*, 2301777.

[S2]	C. Zhou, Y. Yang, J. Wang, Q. Wu, Z. Gu, Y. Zhou, X. Liu, Y. Yang, H. Tang, Q. Ling, L. Wang, J. Zang, *Nat. Commun.* **2021**, *12*, 5072.

[S3]	Y. Yang, J. Wang, L. Wang, Q. Wu, L. Ling, Y. Yang, S. Ning, Y. Xie, Q. Cao, L. Li, J. Liu, Q. Ling, J. Zang, *Sci. Adv.* **2022**, *8*, eabq1456.

[S4]	X. Zhao, Y. Zhou, J. Xu, G. Chen, Y. Fang, T. Tat, X. Xiao, Y. Song, S. Li, J. Chen, *Nat. Commun.* **2021**, *12*, 6755.

[S5]	Y. Sun, W. Zhang, J. Gu, L. Xia, Y. Cao, X. Zhu, H. Wen, S. Ouyang, R. Liu, J. Li, Z. Jiang, D. Cheng, Y. Lv, X. Han, W. Qiu, K. Cai, E. Song, Q. Cao, L. Li, *Nat. Commun.* **2024**, *15*, 1839.

[S6]	Z. Zhang, J. T. Heron, A. Pena‐Francesch, *Adv. Funct. Mater.* **2023**, *33*, 2215248.

[S7]	Y. Zhang, C. Pan, P. Liu, L. Peng, Z. Liu, Y. Li, Q. Wang, T. Wu, Z. Li, C. Majidi, L. Jiang, *Nat. Commun.* **2023**, *14*, 4428.

[S8]	Y. Chi, E. E. Evans, M. R. Clary, F. Qi, H. Sun, S. N. Cantú, C. M. Capodanno, J. B. Tracy, J. Yin, *Sci. Adv.* **2024**, *10*, eadr8421.

[S9]	Y. Lee, F. Koehler, T. Dillon, G. Loke, Y. Kim, J. Marion, M. Antonini, I. C. Garwood, A. Sahasrabudhe, K. Nagao, X. Zhao, Y. Fink, E. T. Roche, P. Anikeeva, *Adv. Mater.* **2023**, *35*, 2301916.

[S10]	Z. Ren, W. Hu, X. Dong, M. Sitti, *Nat. Commun.* **2019**, *10*, 2703.

[S11]	W. Hu, G. Z. Lum, M. Mastrangeli, M. Sitti, *Nature* **2018**, *554*, 81.

[S12]	Y. Dong, L. Wang, N. Xia, Z. Yang, C. Zhang, C. Pan, D. Jin, J. Zhang, C. Majidi, L. Zhang, *Sci. Adv.* **2022**, *8*, eabn8932.

[S13]	S. Nong, Y. Sun, B. Sun, X. Li, Z. Zhu, J. Wu, D. Li, W. Li, S. Zhang, M. Li, *Adv. Funct. Mater.* **2025**, *35*, 2415690.

[S14]	A. C. Karacakol, Y. Alapan, S. O. Demir, M. Sitti, *Nat. Commun.* **2025**, *16*, 2946.

[S15]	N. Arora, V. Chen, A. Cherkasov, Y. Xiang, A. Juhl, P. Buskohl, S. Rudykh, *Adv. Funct. Mater.* **2024**, *34*, 2401077.

[S16]	L. Wang, Y. Chang, S. Wu, R. R. Zhao, W. Chen, *Nat. Commun.* **2023**, *14*, 8516.

[S17]	Y. Kim, H. Yuk, R. Zhao, S. A. Chester, X. Zhao, *Nature* **2018**, *558*, 274.

[S18]	Y. Alapan, A. C. Karacakol, S. N. Guzelhan, I. Isik, M. Sitti, *Sci. Adv.* **2020**, *6*, eabc6414.

[S19]	Y. Sun, L. Wang, Z. Zhu, X. Li, H. Sun, Y. Zhao, C. Peng, J. Liu, S. Zhang, M. Li, *Adv. Mater.* **2023**, *35*, 2302824.
